# PdumBase: a transcriptome database and research tool for Platynereis dumerilii and early development of other metazoans

**DOI:** 10.1186/s12864-018-4987-0

**Published:** 2018-08-16

**Authors:** Hsien-Chao Chou, Natalia Acevedo-Luna, Julie A. Kuhlman, Stephan Q. Schneider

**Affiliations:** 1 Department of Genetics, Developmental and Cell Biology, Iowa State University, 503 Science Hall II, Ames, IA 50011 USA; 2 Present address: Center for Cancer Research, National Institutes of Health, Rockville, MD 20894 USA

**Keywords:** *Platynereis dumerilii*, Early development, Spiralian, Life cycle, Expression profile, Database. Comparative genomics, Evo-devo

## Abstract

**Background:**

The marine polychaete annelid *Platynereis dumerilii* has recently emerged as a prominent organism for the study of development, evolution, stem cells, regeneration, marine ecology, chronobiology and neurobiology within metazoans. Its phylogenetic position within the spiralian/ lophotrochozoan clade, the comparatively high conservation of ancestral features in the *Platynereis* genome, and experimental access to any stage within its life cycle, make *Platynereis* an important model for elucidating the complex regulatory and functional molecular mechanisms governing early development, later organogenesis, and various features of its larval and adult life. High resolution RNA-seq gene expression data obtained from specific developmental stages can be used to dissect early developmental mechanisms. However, the potential for discovery of these mechanisms relies on tools to search, retrieve, and compare genome-wide information within *Platynereis*, and across other metazoan taxa.

**Results:**

To facilitate exploration and discovery by the broader scientific community, we have developed a web-based, searchable online research tool, PdumBase, featuring the first comprehensive transcriptome database for *Platynereis dumerilii* during early stages of development (2 h ~ 14 h). Our database also includes additional stages over the *P. dumerilii* life cycle and provides access to the expression data of 17,213 genes (31,806 transcripts) along with annotation information sourced from Swiss-Prot, Gene Ontology, KEGG pathways, Pfam domains, TmHMM, SingleP, and EggNOG orthology. Expression data for each gene includes the stage, the normalized FPKM, the raw read counts, and information that can be leveraged for statistical analyses of differential gene expression and the construction of genome-wide co-expression networks. In addition, PdumBase offers early stage transcriptome expression data from five further species as a valuable resource for investigators interested in comparing early development in different organisms. To understand conservation of *Platynereis* gene models and to validate gene annotation, most *Platynereis* gene models include a comprehensive phylogenetic analysis across 18 species representing diverse metazoan taxa.

**Conclusions:**

PdumBase represents the first online resource for the early developmental transcriptome of *Platynereis dumerilii*. It serves as a research platform for discovery and exploration of gene expression during early stages, throughout the *Platynereis* life cycle, and enables comparison to other model organisms. PdumBase is freely available at http://pdumbase.gdcb.iastate.edu.

**Electronic supplementary material:**

The online version of this article (10.1186/s12864-018-4987-0) contains supplementary material, which is available to authorized users.

## Background

The annelid *Platynereis dumerilii* is an ideal organism for developmental and comparative studies due to its phylogenetic position as a spiralian/lophotrochozoan, a species-rich but less known third branch of bilateral symmetrical organisms, whose members are instrumental for inferring ancestral bilaterian or urbilaterian features [1–5]. However, in comparison to the other two major bilaterian branches, the deuterostomes that include vertebrates and humans, and the ecdysozoans that include nematodes and arthropods like *C. elegans* and the fruit fly *Drosophila*, respectively, substantially less genetic and molecular studies have been performed in spiralian species. As a spiralian, *Platynereis dumerilii* exhibits intriguing developmental features that include a stereotypic cleavage pattern and invariant cell lineages with predictable cell fates [6–8]. Having invariant cell lineages makes it possible to link gene expression in distinct embryonic cells to later cell progeny and organs in larval and adult stages. Knowing this allows to make predictions, and test hypothesis of how molecular processes regulate the cellular origin and composition of organs and body parts [9, 10]. To date, this powerful property has only been exploited in a limited number of studies [11, 12] but, as better descriptions of later cell lineages and access to stage-specific and cell-specific gene expression data becomes available, its use is expected to increase substantially [13–16]. Additionally, the morphological and genomic attributes exhibited by *Platynereis* have been useful for inferring ancestral gene structures and ancient cell types representative of ancestral bilaterian species [17, 18]. These inferences are based on the findings that *Platynereis* shares many common features with vertebrates, including similar gene expression profiles during the development of the brain, the central nervous system, the eye, appendages, and muscles. These features have been instrumental in developing and testing hypotheses about the urbilaterian body plan and the origin of complex organ systems during animal evolution [3, 4, 19–22]. For these reasons, over the last decade, *Platynereis* has emerged as a powerful spiralian model for comparative genomic analyses, evolutionary developmental biology (evo-devo) studies, stem cells, regeneration, marine ecology, chronobiology and neurobiology [23–27]. *Platynereis* as a laboratory organism boasts an ever-increasing experimental toolkit, expanding the usefulness of this species in identifying and dissecting gene function: during early and late development; during organogenesis; in various cell types; and in neuronal circuits that dictate the circadian and lunar rhythm-controlled swimming behaviors [27–35].

The on average 3 months life cycle of *Platynereis dumerilii* including sexual maturation and mating synchronized by a monthly lunar cycle is well established under laboratory conditions, and contributes to the attractiveness of this annelid spiralian species as a powerful experimental organism [36]. Mating leads to ‘instant’ external fertilization of thousands of eggs, allowing experimental access to thousands of highly synchronously developing embryonic, larval, juvenile, and adult stages that can be used for large scale biochemical and a variety of Omics studies [37].

Early development in *Platynereis* is characterized by a spiral cleavage mode, a series of invariant, stereotypic asymmetric cell divisions stages, generating a spiral arrangement of embryonic cells characterized by vastly different cell sizes and distinct cell fates [6–9]. This early embryonic spiral cleavage phase transitions after six rounds of cell divisions [about 7 h post fertilization (hpf) at 18 °C], to bilateral symmetrical oriented asymmetric cell divisions. The time of development depends drastically on temperature. Features described at each stage refer to embryos developing at controlled temperature of 18 °C. By 14hpf, the continued rapid cell divisions have given rise to a distinctly organized embryo of ~ 330 cells, a pre-trochophore larval stage that emerges from the jelly coat that surrounds the egg, and begins to rotate via a ring of differentiated multi-ciliated cells, the prototroch, and a developing apical organ, a sensory organ at the animal pole [37].

Within the next few days of development *Platynereis* transitions through several free-swimming larval stages (a primitive trochophore at 24hpf, a more elaborate meta-trochophore at 48hpf, and a nectochaeta larvae at 72hpf) [37]. These stages are morphologically characterized by the additions of distinct ciliary structures, an elaboration of the head and trunk region including the establishment of a complex nervous system, larval and adult eyes, and the formation of trunk segments that bear the first pairs of appendages. After 1 week, the now juvenile three segmented young worm switches from a free swimming to a benthic lifestyle. A growth zone at the posterior end continues to add segments and serial appendages throughout juvenile and adult stages while growing to its adult size of ~ 2 in. in length and sexual maturation within 3 months.

The above attributes make *Platynereis* a favorable subject for various high-throughput sequencing (HTS) techniques including but not limited to gene expression profiling though the quantitative analysis of transcriptomes (RNA-seq). RNA-seq provides an unbiased approach to determine the transcriptional inventory for a process, and, by capturing the dynamic temporal expression profiles through the identification of differentially expressed genes between developmental states, enables investigators to gain system-level insights into organismal development [10].

The massive amount of data produced by modern HTS experiments is both a challenge and an opportunity for countless biological discoveries. However, it can only be effectively utilized as a scientific resource in conjunction with dedicated computational pipelines to preprocess, analyze, store, and visualize this information. Well-established algorithms for preprocessing of raw sequencing reads in terms of quality control [38, 39] and de-multiplexing [40, 41] as well as robust approaches for transcriptome assembly [42–44], differential expression analysis [45–47] and functional enrichment studies have been developed. However, as the generated HTS datasets capture a genome-wide transcriptional response, the generated data richness and complexity is often magnitudes higher than the focused interest of the target study. Consequently, much of the data remains unexplored or is not easily accessible to the scientific community. Thus, computational and bioinformatics strategies have to be developed that enable broad, fast, and long-term accessibility of the data, as well as the intuitive ability to easily search, extract, and/or to graphically visualize molecular processes, pathways, and structural composition.

Ideally, such a research tool opens the discovery space and should be capable of efficiently retrieving the information of interest while scaling well with large data sizes. This in turn requires the processed data to be stored in a standardized, queryable, and platform-independent manner such that an application-specific subset of data can efficiently be served to an algorithmic solution built on top of such system. Notably, centralized information systems, such as relational databases, fulfill all of the above requirements by providing storage solutions combined with powerful querying language such as Structured Query Languages (SQL) [48]. These systems can be further combined with modern web technologies to form a graphical user interface capable of representing vast amounts of data in a concise, interactive and platform agnostic manner. Indeed, web-based database solutions have previously been leveraged to represent a large array of biological data over a wide range of organisms [49–51], as well as metadata such as functional annotations [52] and pathway information [53].

For *Platynereis*, several transcriptomic datasets have recently been reported [15, 34, 54], covering various developmental, larval, juvenile, and adult stages during its 3 month long life cycle. However, public accessibility, as well as ease of data analysis is currently limited and requires a priori knowledge of a transcript/gene to be able to retrieve sequence and expression data. This prior knowledge however is often not available, motivating the need for the development of databases, which enable investigators to query transcriptome and expression data based on functional annotation as well as other features. In addition, only a limited number of attempts have been made to link data to other important developmental model species in order to facilitate comparisons of developmental and cell biological modules, a prerequisite for building testable hypothesis in the field of evolutionary and developmental biology.

To close this gap, we developed PdumBase, a comprehensive, stand-alone, and user-friendly web-based resource, offering researchers a sophisticated platform to study molecular function, expression patterns, and relationships between genes at early embryonic stages in *Platynereis dumerilii*. PdumBase comprises transcriptomic data from the first 14 h of development of *Platynereis* capturing the transition from a single fertilized egg to a ~ 330 cells hatched early protrochophore stage. The data in PdumBase represents the first *Platynereis* transcriptome obtained from seven time points of early embryonic development, recently reported by our group [55]. PdumBase also displays data from a novel analysis to construct genome-wide co-expression networks to identify hub genes and genes sharing similar expression patterns in early embryonic stages. Furthermore, PdumBase incorporates transcriptomic data from Conzelmann M. et al. [34] that includes selected time points of later developmental stages including larval, juvenile, and adult stages covering the entire life cycle, as well as sexually mature male and female worms. To facilitate comparative and evo-devo studies PdumBase includes searchable expression data from distinct developmental stages of six other species that were available at the time of database construction: *Strongylocentrotus purpuratus* [56], *Xenopus tropicalis* [57], *Danio rerio* [58], *Ascaris suum* [59], *Nematostella vectensis* [60], and human [61]. The selection of species for comparative analysis of early development, includes a deuterostome invertebrate and vertebrates, an ecdysozoan, a cnidarian, and transcriptional ‘germ layer’ states of early differentiated human stem cells, respectively, an arbitrary yet informative choice for this ‘pilot’ feature of PdumBase that will be expanded in future releases. Finally, to understand how the *Platynereis* gene models are related to genes in other species, and to provide additional validation for the previous gene annotations, a comprehensive phylogenetic analysis that includes 18 selected species, representing different metazoan taxa of various evolutionary distances, including key spiralian species like the annelid *Capitella teleta* and the mollusk *Crassostrea gigas,* is available for each *Platynereis* gene model. Thus, this research tool enables exploration, discovery and hypothesis building to the community at large by providing intuitive and interactive access to genome-wide developmental expression data of early development in *Platynereis.*

## Construction and content

**Fig. 1:**
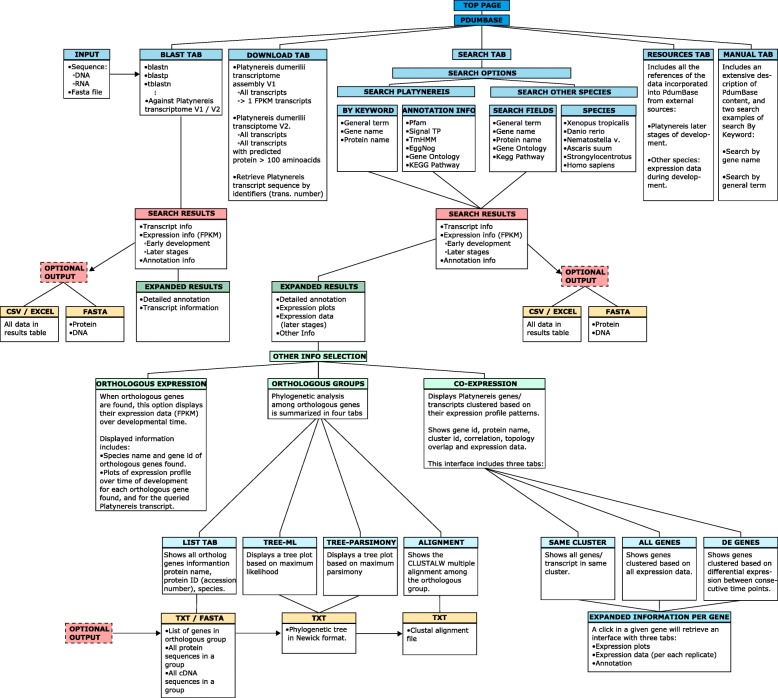
Schematic illustration of the PdumBase sitemap. The content for each tabulator of the PdumBase home interface is summarized in “Tab Boxes” (blue) at the top of the figure.“Search Results” (pink), and “Expanded Results” (dark green) outline data retrieved from different search options. The result interface includes the “Show other info” option; a summary of its content is shown in “Other info selection” box (light green). Expandable results option for. Orthologous groups and Co-expression are also shown (light blue). Optional output summarizes downloadable content as well as file format options (light yellow)

PdumBase is a user-friendly database that provides a platform to easily access and compare expression profiles of *Platynereis* transcripts during early stages of development (2 to 14 hpf), with the option of displaying later stages of development (24 hpf to 3 months, and sexually mature male and female). For each transcript, PdumBase contains detailed annotation, a preliminary phylogeny tree based on orthologous groups, and co-expression networks. Co-expression data can be obtained based on differential gene expression between consecutive time points throughout early development. In addition, the database integrates transcriptomic information of six additional species, giving the option of examining expression data of orthologous genes. Fig. 1 summarizes the content and the organization of data available in PdumBase.

### Transcriptome assembly and annotation pipeline

To create the early reference transcriptome for *Platynereis dumerilii*, we combined the mRNA data of all sequenced libraries (~ 785 million paired-end reads) at 2, 4, 6, 8, 10, 12, and 14 hpf and performed de novo transcriptome assembly using Trinity [62] resulting in a total of 273,087 non-redundant contigs (N50 size: 1466 bp). Detailed methods, characterization and validation on the transcriptome de novo assembly, and the annotation pipeline for gene models have been reported previously [55].

To annotate the assembly, we first identified 28,580 genes (51,260 transcripts) with a predicted open reading frame (ORF) of more than 100 amino acids. These potential protein coding genes were consequently aligned to the Swiss-Prot database [63] and only retained, if the corresponding E-value of the alignment had a value of at least 10e-10 or lower. Using this procedure, a total of 17,213 genes (31,806 transcripts) were matched [55]. Pfam domains, signal peptide cleavage sites and transmembrane helices were also identified and integrated into the database. These were predicted using HMMER [64], signal [65] and tmHMM [66] respectively with default parameters. Finally, Gene Ontology (GO) and eggNOG information were obtained from the Swiss-Prot database [63], whereas associated KEGG pathways were identified by a BLASTP search with an E-value cutoff 10e-10.

### Expression analysis

To build a comprehensive transcriptional profile of early *Platynereis* development, we extracted mRNA from *Platynereis dumerilii* 2, 4, 6, 8, 10, 12, and 14 h post fertilization (hpf), each with two biological replicas, as described [55]. The resulting reads from these samples were subsequently mapped to the assembled transcripts using Bowtie [67]. The read count for each transcript was estimated by RSEM [68]. The trimmed mean of M-values (TMM) [42], normalized FPKM was calculated using the R package “edgeR” [45]. A transcript was considered as “expressed” if its FPKM is larger than 1. This represents an empirically chosen inclusionary cutoff based on our current methods of validation (1) by whole mount in situ hybridization, which is able to detect transcripts above a level of ~ 5 FPKM, and (2) successful amplification of transcripts by PCR from stages with an FPKM of 1 or higher. Whole mount in situ hybridization was chosen as a method of validation to enable us to determine spatial expression of transcripts, and as a method that was sensitive enough to visualize the onset of gene transcription in a single cell, and on two genomic loci within each nucleus [55].

Thus, for expression analysis, 20,977 transcripts were included that fulfilled the criteria of being expressed in at least one stage at an FPKM larger than 1. These transcripts were clustered based on their expression pattern into 32 groups. We obtained the expression dendrogram using the hierarchical clustering algorithm (R function hclust). Since the determination of number of clusters is context-dependent, we first used an automatic tree-cutting algorithm to classify all genes into 32 groups (R function cutree, k = 32). Pairs of groups were then merged as moving up the hierarchy. Each merge was validated by visual inspection of their average expression patterns of all stages. We repeated this step until no qualified merges were found. This process leads to 15 distinct clusters demonstrating distinct expression patterns. For successive time stages, the differentially expressed (DE) genes were determined using edgeR and considered as differentially expressed if the adjusted *p*-value was smaller or equal to 0.001. A co-expression network was also constructed using WGCNA [69], an R package for weighted correlation network analysis. The co-expression network can provide not only the correlation between two genes, but also their topological similarity. This similarity captures how closely related two genes are by examining if they share similar connectivity with other “third party” genes (Additional file 1: Figure S1). In addition to early stage expression data (2 to 14 hpf), we also incorporated 310 million reads of later stage RNA sequencing datasets ranging from 24 h to 3 months, and sexually mature male and female provided by Conzelmann, et al. [34], that were first mapped to our gene models and displayed within the database. The inclusion of this information provides a more comprehensive time series of gene expression profiles at various times throughout the *Platynereis dumerilii* life cycle.

### Comparative transcriptome

To enable comparative studies of gene expression between model organisms during early embryogenesis, we incorporated the expression data from six additional species including *Danio rerio* [58], *Xenopus tropicalis* [57], *Homo sapiens* [61], *Strongylocentrotus purpuratus* [56], *Nematostella vectensis* [60] and *Ascaris suum* [59]. Orthologous groups were identified using OrthoMCL [70] with reciprocal BLASTP search at protein level.

To further assist comparative analyses and to provide annotation validation, we constructed a large scale evolutionary comparison between our gene models with 17 other species in phylogenetically informative positions including *Capitella teleta* [71], *Helobdella robusta* [71], *Lottia gigantea* [71] *Crassostrea gigas* [72], *Daphnia pulex* [71], *Tribolium castaneum* [71]*, Drosophila melanogaster* [50], *Strongylocentrotus purpuratus* [51], *Saccoglossus kowalevskii* [71], *Branchiostoma floridae* [71], *Danio rerio* [73], *Xenopus tropicalis* [71], *Homo sapiens* [73], *Nematostella vectensis* [71], *Amphimedon queenslandica* [71], *Trichoplax adherens* [71], and *Monosiga brevicollis* [71]. A custom OrthoMCL pipeline was used to identify a total of 40,206 homologous groups out of which 32,482 groups have at least two species. A pairwise BLASP search with cutoff 1e-05 and 50% identity was performed. The orthologous relationship was established only if genes are the reciprocal best hits for any two species. For each *Platynereis dumerilii* gene having orthologous genes, a phylogenetic tree was created using RAxML [74]. Both, maximum likelihood and parsimony trees can be accessed in PdumBase. The multiple alignment for each orthologous group is constructed using CLUSTALW [75].

## Utility and discussion

PdumBase provides a comprehensive, versatile online tool to investigate stage specific transcriptional inputs during embryogenesis and throughout the life cycle of the annelid *Platynereis dumerilii* and other selected species. Users can search the genes of interest and examine their functional annotation, expression profiles, co-expression networks, and use the genes for comparative analyses. In addition to the search module, PdumBase also offers the option of downloading the queried data and searching the transcriptome using BLAST. Here we describe the interface and functions of these modules.

### Annotation search

**Fig. 2:**
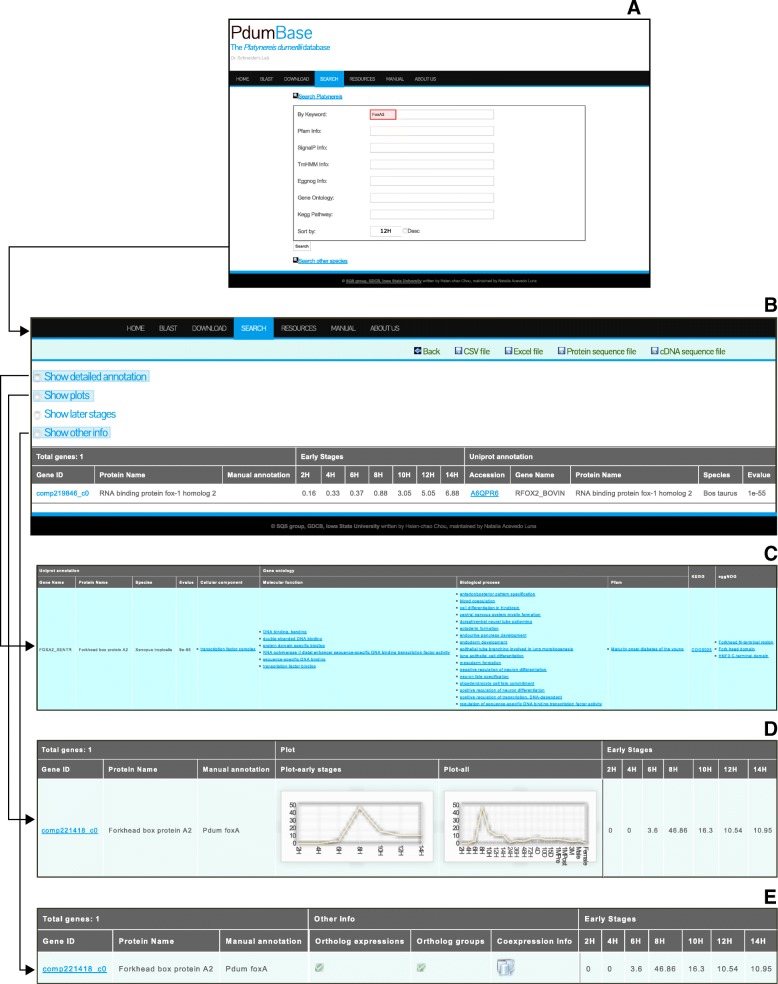
PdumBase search interface and expandable search result options. **a** PdumBase Search interface. An example under Functional Annotation on “Keyword search” field is shown. **b** The PdumBase Results interface displays the expression data at different stages of development for each identified gene. **c** Screen capture of the interface obtained when the option “Show detailed annotation” is selected from the Results screen. Detailed annotation includes Gene Ontology, KEGG, EggNOG, and more. **d** The results interface when the option “Show plots” is selected. This option displays the expression data (in FPKM) plotted over time of development for each gene found. **e** Result interface with the “Show other info” option selected. Clicking on the Green checkmark icon will redirect to a new tab with expanded search results for: “Ortholog Expression”, “Ortholog groups”, and “Co-expression”

To facilitate utility of PdumBase for a broad range of research questions, users may want to mine data using a variety of search parameters. This versatile accessibility to retrieve genes of interest is provided through a powerful search engine allowing users to query specific keywords covering a large array of functional annotation categories. These include gene ID, protein name, gene ontology, Pfam, KEGG pathways, eggnog, SignalP and tmHMM information. The search results are displayed as an expression profile of the matched genes as well as their functional annotation information. By default, the results page displays the Swiss-Prot annotation based on protein level BLAST search, and the expression data for early stages (2  to 14 hpf). Users can expand this table to include expression profiles of later stages (by checking the “Show later stages” box), detailed annotation (by selecting the “Show detailed annotation” box) and other advanced information by selecting “Show plots” and “Show other info” in the interface (Fig. 2). More detailed annotation can be obtained from the interface with links to the gene ontology, Pfam, KEGG pathways, EggNOG, SignalP and tmHMM information of the corresponding genes. Links to external data sources are also provided. Where available, the links to additional pages will be shown, including the expression of orthologous genes whereas phylogenetic analysis and co-expression network information can be obtained by clicking “Show other info” (Fig. 2).

### Sequence similarity search

In addition to searching using keywords, PdumBase also supports sequence-based queries via the BLAST search page. Users can input either nucleotide or protein sequence. The search options for BLAST, including the E-value, the number of alignments, and the type of search are customizable. The search results return the expression as well as the functional annotation information for the matched sequence.

Additional properties of each gene such as (i) isoform level data and corresponding plots, (ii) replicate information including all biological and technical replicates, (iii) the raw read count information as estimated by RSEM, and (iv) detailed annotation information (Additional file 2: Figure S2) can also be accessed from the gene ID page. While the main results page displays information on the best hit for genes in *Platynereis*, the detailed annotation information provides information regarding the source of the annotation.

### Comparative analysis

For investigators interested in comparative studies, PdumBase also provides a searchable interface for an additional six species including *Danio rerio*, *Xenopus tropicalis*, *Homo sapiens*, *Nematostella vectensis*, *Strongylocentrotus purpuratus,* and *Ascaris suum*.

**Fig. 3:**
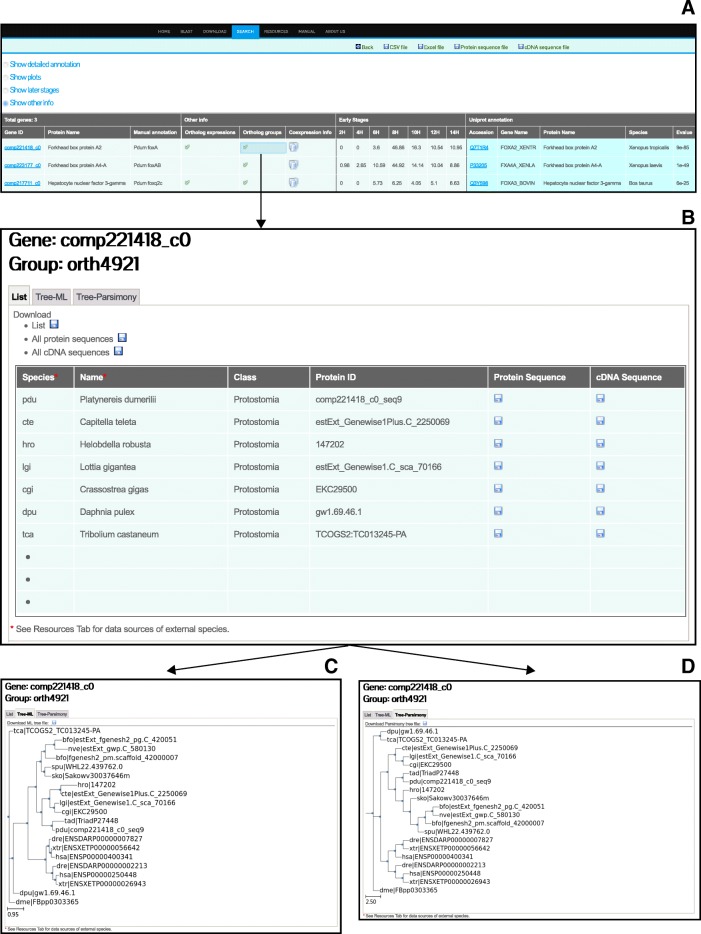
PdumBase expandable results option. **a** PdumBase results interface displaying the options under “Show Other Info”. Click on the green check mark will open a new tab. **b** Interface retrieved when the green check mark icon under “Ortholog group” is selected. The resulting interface displays for the particular *Platynereis* gene under search, all the species in which an orthologous gene was found. The protein and/or cDNA sequences can be downloaded individually for each orthologous gene, or for the complete set shown in the result table. **c** and **d** Display Phylogenetic trees of the orthologous groups: ML tree (**c**) and Tree- Parsimony (**d**)

Through “Show other info” in the search result page, users can link to comparative information provided under the “Ortholog expressions” column if orthologous genes were identified through our automated approach (see above). The developmental expression profiles for all the orthologous genes are also provided in a single window to facilitate easy comparison of orthologous expression patterns. To further facilitate comparative studies and to enable additional validation for the annotations, PdumBase displays the phylogenetic analyses for each *P. dumerilii* gene with homologous counterparts across 18 species. The “Orthologous groups” page includes four tabs: (i) a list of information related to all orthologous genes including the protein name, protein ID (accession number), species, species class and the links for downloading their protein or cDNA sequences, (ii) a tree plot based on maximum likelihood, (iii) a tree plot based on maximum parsimony, and (iv) a CLUSTALW multiple alignment report. The tree files, alignment report and all the protein/cDNA sequences are downloadable (Fig. 3).

### Co-expression information

Expression patterns shared by multiple genes may indicate the function in a similar biological process. By clicking on corresponding entry in the “Co-expression Info” column, PdumBase provides co-expression information that may reveal the potential interaction among a set of genes. This page is tabulated into three categories, namely “The same cluster”, “All”, and “DE genes”. Genes found within the same cluster by clustering analysis are shown in the first tab whereas the second tab includes every developmental gene expression profile independent of the cluster analysis. The co-expression network also provides the topological overlap information, which can be used to examine the similarity of interaction with all other genes in the network. The “DE genes” tab shows the co- expressed genes between each adjacent developmental stage for both, up-regulated and down-regulated genes.

### Database implementation

All data content is stored in a MySQL database. The web interface was implemented using PHP and the Smarty template engine. The business logics and presentation are separated by employing the model-view-controller design pattern. A centralized controller is used to coordinate the client requests and generate the corresponding views. All plots are either generated by server-side R scripts or client-side JavaScripts. In addition, a BLAST database was constructed for the transcriptome assembly using NCBI BLAST.

### Additional features

#### Data export

The annotation and BLAST search results are all downloadable in text format (CSV) or as an Excel sheet. In addition, all assembled transcripts; annotation, expression data and differentially expressed genes are also available for download on our website. The raw Illumina reads are available upon request.

#### Manual

A manual providing a detailed description of all the features and how to access them is available on the PdumBase web page http://pdumbase.gdcb.iastate.edu/platynereis/controller.php?action=manual, as well as in Additional file 3: PDF File 1.

## Conclusions

PdumBase offers a user-friendly platform for researchers to study the regulatory landscape for a spiral-cleaving embryo with an emphasis on early developmental stages of *Platynereis dumerilii,* and selected later stages throughout its life cycle. It provides researchers with an online tool for fast, dynamic, retrieval of expression patterns during early stages of development and life cycle. Furthermore, the large-scale data sets and comparative analyses offer valuable information for the studies of molecular dynamics and evolutionary aspects of the *P. dumerilii* transcriptome in comparison to other model systems. It represents one of the first attempts to integrate and harness developmental expression profiles from various species in combination with phylogeny based annotations offering a versatile online research tool to discover and investigate various aspects of animal evolution and development.

Future expansions for PdumBase may include genome-wide expression profiles after experimental manipulations, single cell transcriptomic data for early stages of development, and genomic information of regulatory regions to provide further entry points for promoter and network analysis. Further database subdivisions could display more detailed and/or manually curated aspect of early development e.g. asymmetric cell division, distinct pathways, or the emergence of distinct cell lineages and cell types. Additionally, we consider the inclusion of images of gene expression data as a possible and valuable future expansion. Further PdumBase expansions may include an interactive visualization of clustered genes based on expression profile. Such tool would enable the user to fine tune the cluster parameters while simultaneously visualizing the change in cluster composition allowing to user to examine different sets of genes at a time.

As such, PdumBase can be seen as a prototype for an online research tool to make any large-scale genome-wide data set quickly accessible to researchers without requiring prior expertise in bioinformatics, showcasing how valuable and extensive transcriptional data set can be made accessible for community wide data mining. The versatility and variety of search options enable a wider range of research questions to be investigated both within a single laboratory and across the scientific community. The creation of such a database opens the genome-wide discovery space of data sets to the entire greater scientific community.

## Additional files

Additional file 1: **Figure S1.** Expandable results from PdumBase Result Interface: Co-expression **A**. The Result Interface with the option “Show other info” selected. **B**. Co-expression information interface under the tabulator “The same cluster” displays all the transcripts/genes in the same cluster of a given component. Shown are protein name, correlation, topology overlap, and expression data. **C**. Co-expression information interface under the tabulator “All” displays all genes sorted by the ranking according to correlation score. **D**. Co-expression information interface under the tabulator “DE genes” displays differentially expressed genes between consecutive time points. (PDF 397 kb)

Additional file 2: **Figure S2.** Expandable results from PdumBase Result Interface: Gene models **A**. The Result Interface allows to click on each gene found, expanding its expression and annotation information. **B**. Shows the interface obtained when clicking on a Gene ID from the result table. Plots of expression data over time of development are shown (early stages and all stages are plotted) Expression plot includes data of all isoforms found for a gene. **C**. Expression Data tab. This tab displays FPKM and Raw counts for a given gene and its possible isoforms. **D**. Annotation Tab. This interface retrieves detailed annotation information. (PDF 502 kb)

Additional file 3: PDF File 1. PdumBase Manual. A description of PdumBase content with detailed instructions on how to use all its features. (PDF 4093 kb)

## Abbreviations

BLAST: Basic Local Alignment Search Tool
FPKM: Fragments Per Kilobase per Million mapped reads
GO: Gene ontology
hpf: Hours post fertilization
KEGG: Kyoto Encyclopedia of Genes and Genomes
ORF: Open reading frame
TMM: Trimmed Mean of M-values
