# Supplementary material for: PdumBase: a transcriptome database and research tool for Platynereis dumerilii and early development of other metazoans
Source: BMC Genomics. 2018 Aug 16;19:618. doi: 10.1186/s12864-018-4987-0 (PMC6097317; doi:10.1186/s12864-018-4987-0)
Supplement: Supplementary file 1 — Figure S1. Expandable results from PdumBase Result Interface: Co-expression A. The Result Interface with the option “Show other info” selected. B. Co-expression information interface under the tabulator “The same cluster” displays all the transcripts/genes in the same cluster of a given component. Shown are protein name, correlation, topology overlap, and expression data. C. Co-expression information interface under the tabulator “All” displays all genes sorted by the ranking according to correlation score. D. Co-expression information interface under the tabulator “DE genes” displays differentially expressed genes between consecutive time points. (PDF 397 kb) [file 12864_2018_4987_MOESM1_ESM.pdf]

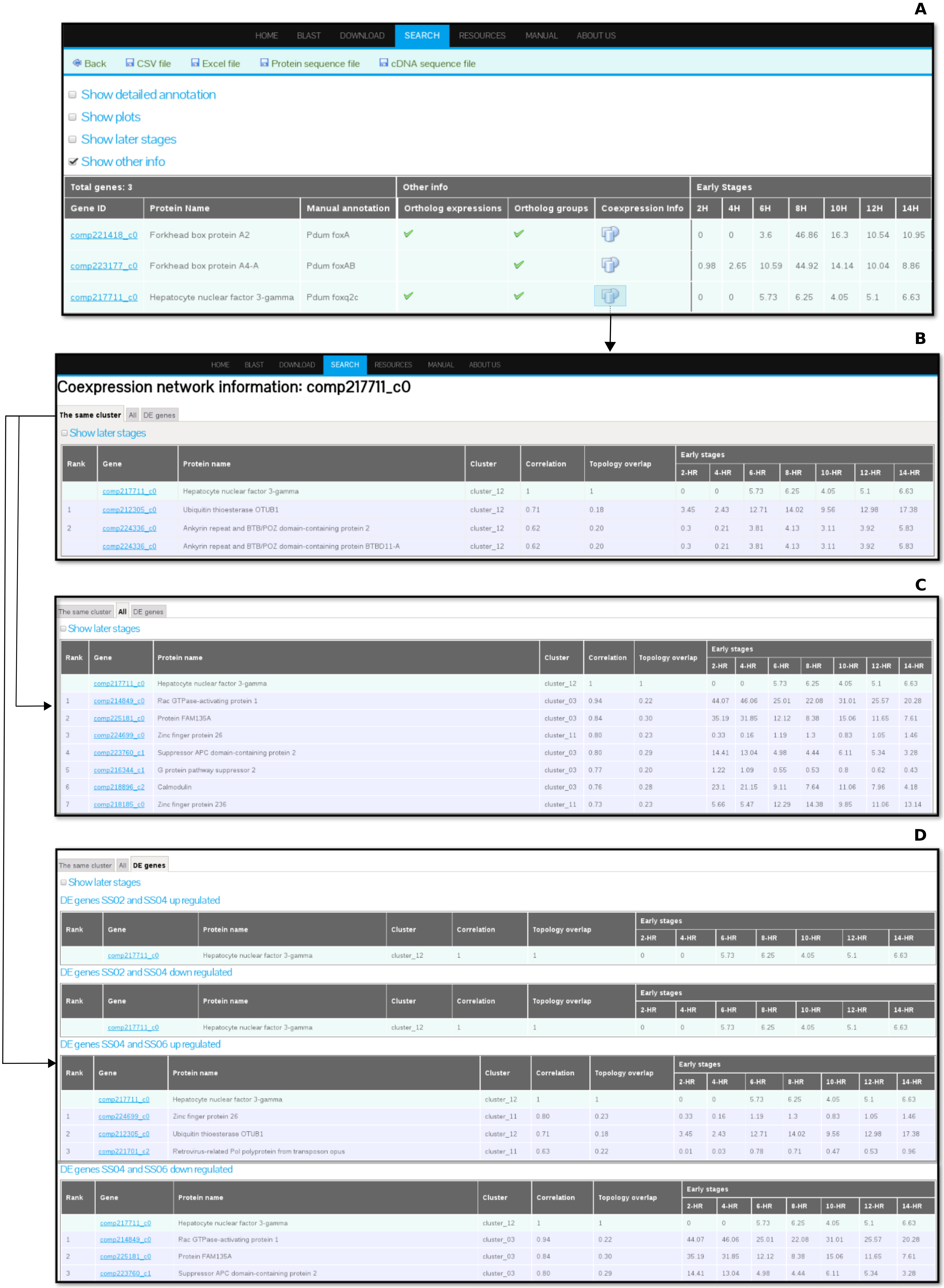

A

HOME | BLAST | DOWNLOAD | SEARCH | RESOURCES | MANUAL | ABOUT US

Back | CSV file | Excel file | Protein sequence file | cDNA sequence file

- [ ] Show detailed annotation
- [ ] Show plots
- [ ] Show later stages
- [x] Show other info

Total genes: 3			Other info			Early Stages						
Gene ID	Protein Name	Manual annotation	Ortholog expressions	Ortholog groups	Coexpression Info	2H	4H	6H	8H	10H	12H	14H
comp221418_c0	Forkhead box protein A2	Pdum foxA	✔	✔		0	0	3.6	46.86	16.3	10.54	10.95
comp223177_c0	Forkhead box protein A4-A	Pdum foxAB		✔		0.98	2.65	10.59	44.92	14.14	10.04	8.86
comp217711_c0	Hepatocyte nuclear factor 3-gamma	Pdum foxq2c	✔	✔		0	0	5.73	6.25	4.05	5.1	6.63

B

HOME | BLAST | DOWNLOAD | SEARCH | RESOURCES | MANUAL | ABOUT US

Coexpression network information: comp217711_c0

The same cluster | All | DE genes

- [ ] Show later stages

Rank	Gene	Protein name	Cluster	Correlation	Topology overlap	Early stages						
						2-HR	4-HR	6-HR	8-HR	10-HR	12-HR	14-HR
	comp217711_c0	Hepatocyte nuclear factor 3-gamma	cluster_12	1	1	0	0	5.73	6.25	4.05	5.1	6.63
1	comp212305_c0	Ubiquitin thioesterase OTUB1	cluster_12	0.71	0.18	3.45	2.43	12.71	14.02	9.56	12.98	17.38
2	comp224336_c0	Ankyrin repeat and BTB/POZ domain-containing protein 2	cluster_12	0.62	0.20	0.3	0.21	3.81	4.13	3.11	3.92	5.83
	comp224336_c0	Ankyrin repeat and BTB/POZ domain-containing protein BTBD11-A	cluster_12	0.62	0.20	0.3	0.21	3.81	4.13	3.11	3.92	5.83

C

The same cluster | All | DE genes

- [ ] Show later stages

Rank	Gene	Protein name	Cluster	Correlation	Topology overlap	Early stages						
						2-HR	4-HR	6-HR	8-HR	10-HR	12-HR	14-HR
	comp217711_c0	Hepatocyte nuclear factor 3-gamma	cluster_12	1	1	0	0	5.73	6.25	4.05	5.1	6.63
1	comp214849_c0	Rac GTPase-activating protein 1	cluster_03	0.94	0.22	44.07	46.06	25.01	22.08	31.01	25.57	20.28
2	comp225181_c0	Protein FAM135A	cluster_03	0.84	0.30	35.19	31.85	12.12	8.38	15.06	11.65	7.61
3	comp224699_c0	Zinc finger protein 26	cluster_11	0.80	0.23	0.33	0.16	1.19	1.3	0.83	1.05	1.46
4	comp223760_c1	Suppressor APC domain-containing protein 2	cluster_03	0.80	0.29	14.41	13.04	4.98	4.44	6.11	5.34	3.28
5	comp216344_c1	G protein pathway suppressor 2	cluster_03	0.77	0.20	1.22	1.09	0.55	0.53	0.8	0.62	0.43
6	comp218896_c2	Calmodulin	cluster_03	0.76	0.28	23.1	21.15	9.11	7.64	11.06	7.96	4.18
7	comp218185_c0	Zinc finger protein 236	cluster_11	0.73	0.23	5.66	5.47	12.29	14.38	9.85	11.06	13.14

D

The same cluster | All | DE genes

- [ ] Show later stages

DE genes SS02 and SS04 up regulated

Rank	Gene	Protein name	Cluster	Correlation	Topology overlap	Early stages						
						2-HR	4-HR	6-HR	8-HR	10-HR	12-HR	14-HR
	comp217711_c0	Hepatocyte nuclear factor 3-gamma	cluster_12	1	1	0	0	5.73	6.25	4.05	5.1	6.63

DE genes SS02 and SS04 down regulated

Rank	Gene	Protein name	Cluster	Correlation	Topology overlap	Early stages						
						2-HR	4-HR	6-HR	8-HR	10-HR	12-HR	14-HR
	comp217711_c0	Hepatocyte nuclear factor 3-gamma	cluster_12	1	1	0	0	5.73	6.25	4.05	5.1	6.63

DE genes SS04 and SS06 up regulated

Rank	Gene	Protein name	Cluster	Correlation	Topology overlap	Early stages						
						2-HR	4-HR	6-HR	8-HR	10-HR	12-HR	14-HR
	comp217711_c0	Hepatocyte nuclear factor 3-gamma	cluster_12	1	1	0	0	5.73	6.25	4.05	5.1	6.63
1	comp224699_c0	Zinc finger protein 26	cluster_11	0.80	0.23	0.33	0.16	1.19	1.3	0.83	1.05	1.46
2	comp212305_c0	Ubiquitin thioesterase OTUB1	cluster_12	0.71	0.18	3.45	2.43	12.71	14.02	9.56	12.98	17.38
3	comp221701_c2	Retrovirus-related Pol polyprotein from transposon opus	cluster_11	0.63	0.22	0.01	0.03	0.78	0.71	0.47	0.53	0.96

DE genes SS04 and SS06 down regulated

Rank	Gene	Protein name	Cluster	Correlation	Topology overlap	Early stages						
						2-HR	4-HR	6-HR	8-HR	10-HR	12-HR	14-HR
	comp217711_c0	Hepatocyte nuclear factor 3-gamma	cluster_12	1	1	0	0	5.73	6.25	4.05	5.1	6.63
1	comp214849_c0	Rac GTPase-activating protein 1	cluster_03	0.94	0.22	44.07	46.06	25.01	22.08	31.01	25.57	20.28
2	comp225181_c0	Protein FAM135A	cluster_03	0.84	0.30	35.19	31.85	12.12	8.38	15.06	11.65	7.61
3	comp223760_c1	Suppressor APC domain-containing protein 2	cluster_03	0.80	0.29	14.41	13.04	4.98	4.44	6.11	5.34	3.28
