# Supplementary material for: PdumBase: a transcriptome database and research tool for Platynereis dumerilii and early development of other metazoans
Source: BMC Genomics. 2018 Aug 16;19:618. doi: 10.1186/s12864-018-4987-0 (PMC6097317; doi:10.1186/s12864-018-4987-0)
Supplement: Supplementary file 2 — Figure S2. Expandable results from PdumBase Result Interface: Gene models A. The Result Interface allows to click on each gene found, expanding its expression and annotation information. B. Shows the interface obtained when clicking on a Gene ID from the result table. Plots of expression data over time of development are shown (early stages and all stages are plotted) Expression plot includes data of all isoforms found for a gene. C. Expression Data tab. This tab displays FPKM and Raw counts for a given gene and its possible isoforms. D. Annotation Tab. This interface retrieves detailed annotation information. (PDF 502 kb) [file 12864_2018_4987_MOESM2_ESM.pdf]

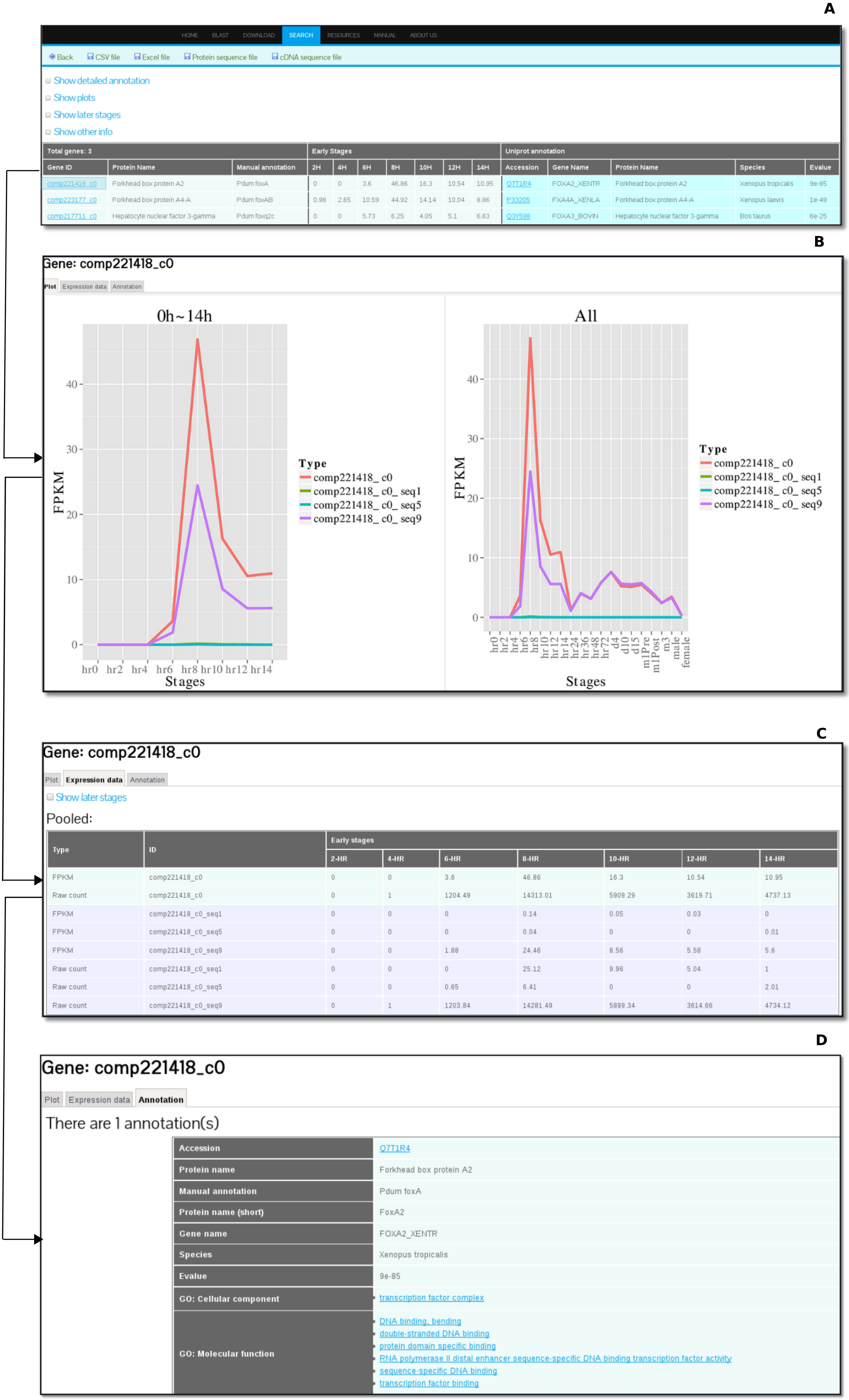

A
HOME BLAST DOWNLOAD SEARCH RESOURCES MANUAL ABOUT US
Back CSV file Excel file Protein sequence file cDNA sequence file
Show detailed annotation
Show plots
Show later stages
Show other info
Total genes: 3
Early Stages
Uniprot annotation
Gene ID | Protein Name | Manual annotation | 2H | 4H | 6H | 8H | 10H | 12H | 14H | Accession | Gene Name | Protein Name | Species | Evalue
comp221418_c0 | Forkhead box protein A2 | Pdum foxA | 0 | 0 | 3.6 | 46.86 | 16.3 | 10.54 | 10.95 | Q7T1R4 | FOXA2_XENTR | Forkhead box protein A2 | Xenopus tropicalis | 9e-85
comp223177_c0 | Forkhead box protein A4-A | Pdum foxAB | 0.98 | 2.65 | 10.59 | 44.92 | 14.14 | 10.04 | 8.86 | P33205 | FXA4A_XENLA | Forkhead box protein A4-A | Xenopus laevis | 1e-49
comp217711_c0 | Hepatocyte nuclear factor 3-gamma | Pdum foxq2c | 0 | 0 | 5.73 | 6.25 | 4.05 | 5.1 | 6.63 | Q3Y598 | FOXA3_BOVIN | Hepatocyte nuclear factor 3-gamma | Bos taurus | 6e-25
B
Gene: comp221418_c0
Plot Expression data Annotation
0h~14h
All
FPKM
Stages
hr0 hr2 hr4 hr6 hr8 hr10 hr12 hr14
hr0 hr2 hr4 hr6 hr8 hr10 hr12 hr14 hr24 hr36 hr48 hr72 d4 d10 d15 m1Pre m1Post m3 male female
Type
comp221418_c0
comp221418_c0_seq1
comp221418_c0_seq5
comp221418_c0_seq9
C
Gene: comp221418_c0
Plot Expression data Annotation
Show later stages
Pooled:
Type | ID | Early stages 2-HR | 4-HR | 6-HR | 8-HR | 10-HR | 12-HR | 14-HR
FPKM | comp221418_c0 | 0 | 0 | 3.6 | 46.86 | 16.3 | 10.54 | 10.95
Raw count | comp221418_c0 | 0 | 1 | 1204.49 | 14313.01 | 5909.29 | 3619.71 | 4737.13
FPKM | comp221418_c0_seq1 | 0 | 0 | 0 | 0.14 | 0.05 | 0.03 | 0
FPKM | comp221418_c0_seq5 | 0 | 0 | 0 | 0.04 | 0 | 0 | 0.01
FPKM | comp221418_c0_seq9 | 0 | 0 | 1.88 | 24.46 | 8.56 | 5.58 | 5.6
Raw count | comp221418_c0_seq1 | 0 | 0 | 0 | 25.12 | 9.96 | 5.04 | 1
Raw count | comp221418_c0_seq5 | 0 | 0 | 0.65 | 6.41 | 0 | 0 | 2.01
Raw count | comp221418_c0_seq9 | 0 | 1 | 1203.84 | 14281.49 | 5899.34 | 3614.66 | 4734.12
D
Gene: comp221418_c0
Plot Expression data Annotation
There are 1 annotation(s)
Accession: Q7T1R4
Protein name: Forkhead box protein A2
Manual annotation: Pdum foxA
Protein name (short): FoxA2
Gene name: FOXA2_XENTR
Species: Xenopus tropicalis
Evalue: 9e-85
GO: Cellular component: transcription factor complex
GO: Molecular function: DNA binding, bending
double-stranded DNA binding
protein domain specific binding
RNA polymerase II distal enhancer sequence-specific DNA binding transcription factor activity
sequence-specific DNA binding
transcription factor binding
