# Supplementary material for: PdumBase: a transcriptome database and research tool for Platynereis dumerilii and early development of other metazoans
Source: BMC Genomics. 2018 Aug 16;19:618. doi: 10.1186/s12864-018-4987-0 (PMC6097317; doi:10.1186/s12864-018-4987-0)
Supplement: Supplementary file 3 — PDF File 1. PdumBase Manual. A description of PdumBase content with detailed instructions on how to use all its features. (PDF 4093 kb) [file 12864_2018_4987_MOESM3_ESM.pdf]

PdumBase

The *Platynereis dumerilii* Database

Spiralian embryogenesis at your finger tips

An introductory tutorial into its content and features.

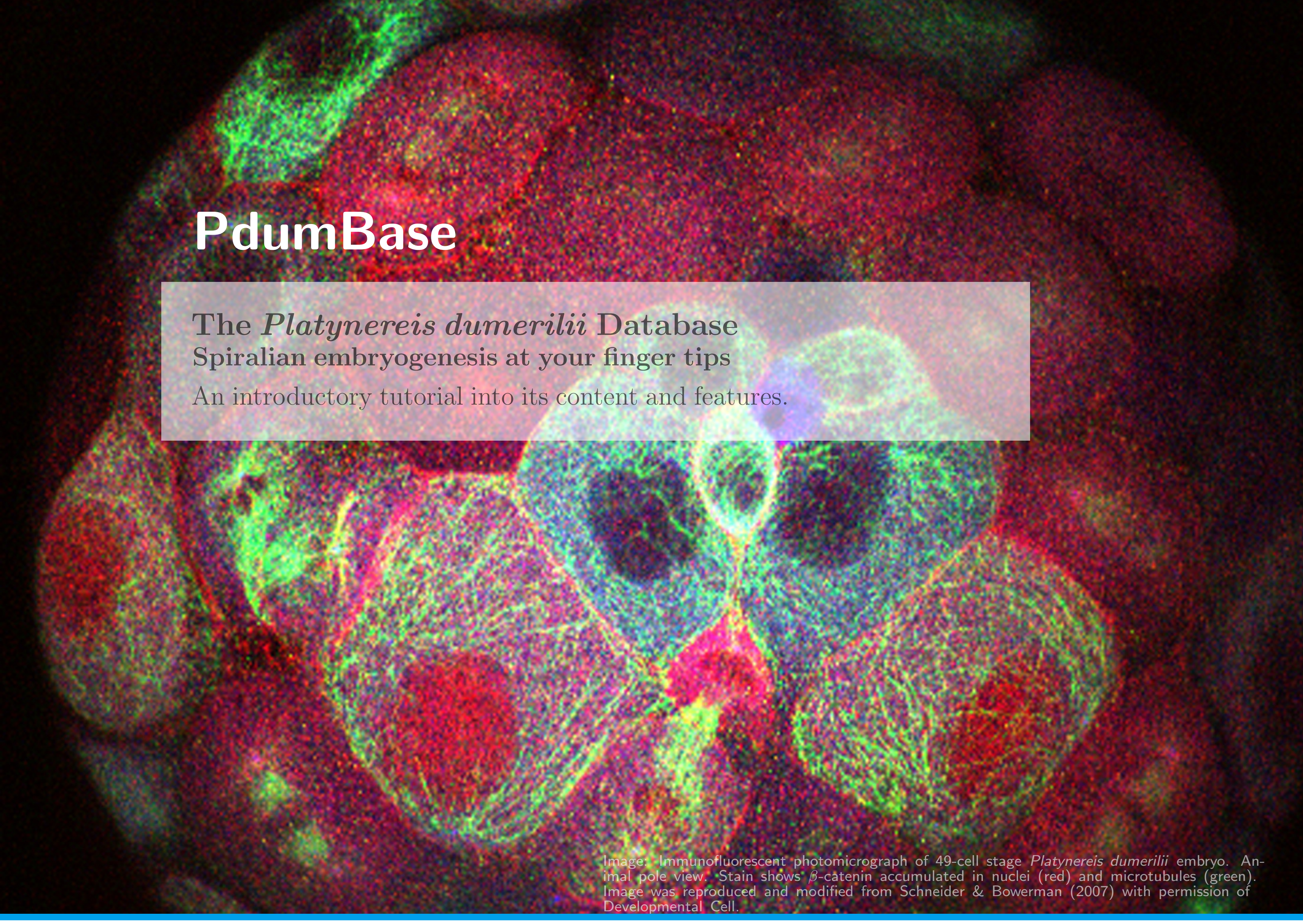

Image: Immunofluorescent photomicrograph of 49-cell stage *Platynereis dumerilii* embryo. Animal pole view. Stain shows β-catenin accumulated in nuclei (red) and microtubules (green). Image was reproduced and modified from Schneider & Bowerman (2007) with permission of Developmental Cell.

The Schneider lab *Platynereis dumerilii* online database: PdumBase, provides a comprehensive, versatile online tool to investigate stage specific transcriptional inputs during embryogenesis and during the life cycle of the annelid *Platynereis dumerilii* and other selected species (e.g. *Danio rerio*, *Xenopus tropicalis*, *Nematostella vectensis*, *Strongylocentrotus purpuratus*).

This document provides a brief description of the database content and a detailed guide on how to browse its data thorough exemplary searches. The tutorial is intended as a motivational introduction while exploring and trying out the features PdumBase has to offer as an online resource to integrate and visualize our data and findings.

1

Database Content

In the following, the database content as well as its structure is explained. First, the details of the raw RNA-Seq data sets are highlighted, followed by an introduction into their corresponding expression data and associated annotation profiles. Furthermore the gene expression profiling features of this software are introduced, followed by an introduction into *Platynereis* specific coexpression networks as well as their comparative transcriptome data.

1. RNA-Seq Data sets

Platynereis dumerilii Normal Development Data Set

The Normal Development Data Set is comprised of two sources of information each of which corresponding to different stages of development *Platynereis dumerilii* at specific time points.

- **Early stages data set:** RNA-seq data generated by Schneider lab

Description: This data corresponds to the first comprehensive transcriptome draft during early development in *Platynereis dumerilii* using the de novo assembly strategy. We performed mRNA deep sequencing of distinct stages using the Illumina HiSeq sequencing system with read lengths of 75bp to 100bp.

Time points: 0, 2, 4, 6, 8, 10, 12, 14 hours post fertilization (hpf). Each stage has two biological replicates. The depth of these libraries ranges from 40 to 120 million paired-end reads (see Table 1).

Table 1: Time points from Early Stages data set

Time (hpf)	Description	Time (hpf)	Description
0	Unfertilized egg	8	$\sim$ 80-cell
2	Zygote	10	$\sim$ 140-cell
4	$\sim$ 8-cell	12	$\sim$ 220-cell
6	$\sim$ 30-cell	14	$\sim$ 330-cell

Assembly: All the biological replicates, which contain about 1.5 billion reads, were assembled into 357,961 transcripts in a genome independent manner. Due to alternative splicing events, out of the total transcripts assembled, 193,310 belong to genes.

Time points: This data set consist of 10 time points from 24 hours post fertilization to 3 months old adults. This set also includes female and male RNA-seq samples. There are no biological replicates (Table 2).

Table 2: Late Stages included in data set. Time points are shown in hours post fertilization (hpf), days (d) and months (M).

Time	Description
24 (hpf)	Early trochophore larvae
36 (hpf)	Mid trochophore larvae
48 (hpf)	Early metatrochophore larvae
72 (hpf)	Early nectochaete larvae
4 d	Mid nectochaete larvae
10 d	Errant juvenile
15 d	3-segmented errant juvenile
1 Mpre	
1 Mpost	
3 M	Adult
Male	Sexually mature adult
Female	Sexually mature adult

2. Expression data

The **PdumBase** web interface displays the mean FPKM (fragments per kilobase per million reads mapped) as the default measurement of gene expression. The FPKM for each replicate was obtained by normalizing the total number of mappable reads with the corresponding transcript length. A transcript or gene is considered as expressed if its FPKM is ≥ 1. Furthermore, the FPKM for each stage was obtained by combining the replicates into a single set.
The result search page displays the mean FPKM values as the default measurement of gene expression (see Figure 1). However, FPKM values from individual samples, as well as the raw counts of each transcript can also be retrieved by clicking on the **"Expression data"** tab after selecting a particular transcript of interest (Figure 2). For more information we refer the reader to the *Tutorial Example* Section.

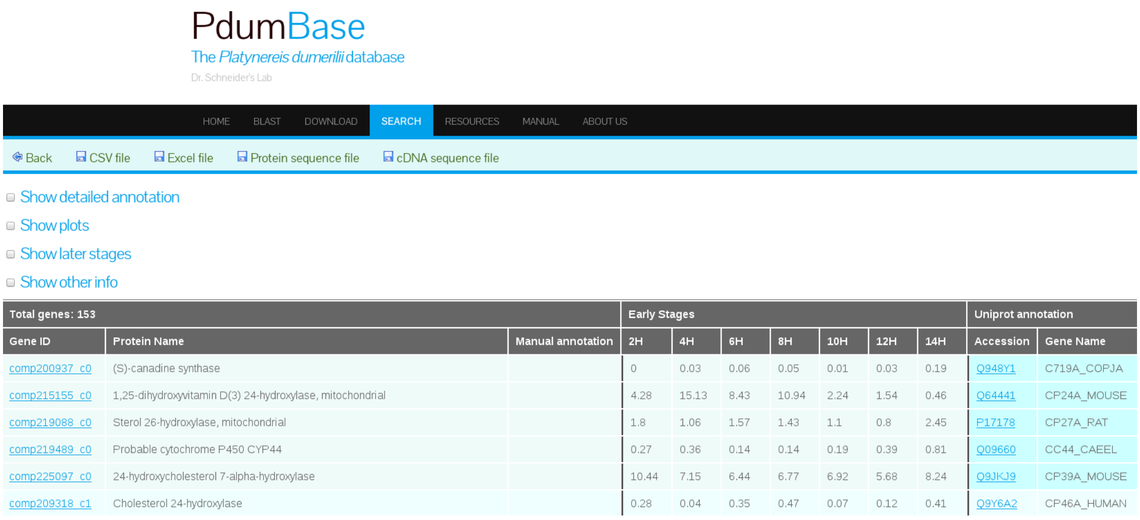

Total genes: 153			Early Stages							Uniprot annotation	
Gene ID	Protein Name	Manual annotation	2H	4H	6H	8H	10H	12H	14H	Accession	Gene Name
comp200937_c0	(S)-canadine synthase		0	0.03	0.06	0.05	0.01	0.03	0.19	Q948Y1	C719A_COPJA
comp215155_c0	1,25-dihydroxyvitamin D(3) 24-hydroxylase, mitochondrial		4.28	15.13	8.43	10.94	2.24	1.54	0.46	Q64441	CP24A_MOUSE
comp219088_c0	Sterol 26-hydroxylase, mitochondrial		1.8	1.06	1.57	1.43	1.1	0.8	2.45	P17178	CP27A_RAT
comp219489_c0	Probable cytochrome P450 CYP44		0.27	0.36	0.14	0.14	0.19	0.39	0.81	Q09660	CC44_CAEEL
comp225097_c0	24-hydroxycholesterol 7-alpha-hydroxylase		10.44	7.15	6.44	6.77	6.92	5.68	8.24	Q9JKJ9	CP39A_MOUSE
comp209318_c1	Cholesterol 24-hydroxylase		0.28	0.04	0.35	0.47	0.07	0.12	0.41	Q9Y6A2	CP46A_HUMAN

Figure 1: **PdumBase Search result interface** displays mean FPKM as measurement of absolute expression

3. Annotation

This section is concerned with describing the different annotations, how these were sourced from external databases for convenient browsing and data exploration specific to *Platynereis dumerilii*.

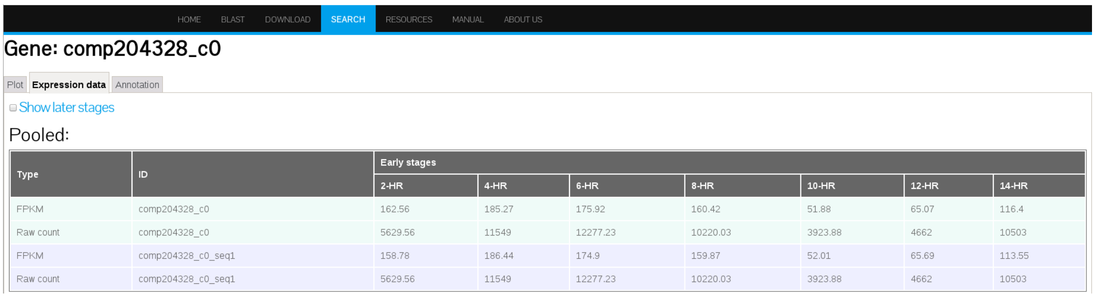

Type	ID	Early stages						
		2-HR	4-HR	6-HR	8-HR	10-HR	12-HR	14-HR
FPKM	comp204328_c0	162.56	185.27	175.92	160.42	51.88	65.07	116.4
Raw count	comp204328_c0	5629.56	11549	12277.23	10220.03	3923.88	4662	10503
FPKM	comp204328_c0_seq1	158.78	186.44	174.9	159.87	52.01	65.69	113.55
Raw count	comp204328_c0_seq1	5629.56	11549	12277.23	10220.03	3923.88	4662	10503

(a)

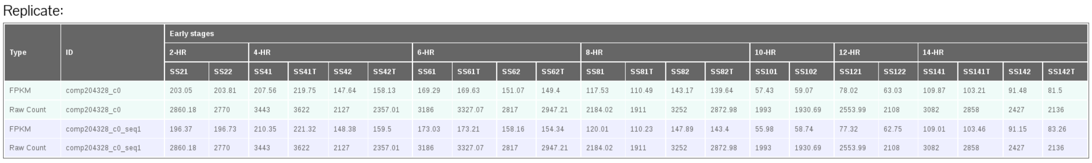

Type	ID	Early stages																					
		2-HR		4-HR				6-HR				8-HR				10-HR		12-HR		14-HR			
		SS21	SS22	SS41	SS41T	SS42	SS42T	SS61	SS61T	SS62	SS62T	SS81	SS81T	SS82	SS82T	SS101	SS102	SS121	SS122	SS141	SS141T	SS142	SS142T
FPKM	comp204328_c0	203.05	203.81	207.56	219.75	147.64	158.13	169.29	169.63	151.07	149.4	117.53	110.49	143.17	139.64	57.43	59.07	78.02	63.03	109.87	103.21	91.48	81.5
Raw Count	comp204328_c0	2860.18	2770	3443	3622	2127	2357.01	3186	3327.07	2817	2947.21	2184.02	1911	3252	2872.98	1993	1930.69	2553.99	2108	3082	2858	2427	2136
FPKM	comp204328_c0_seq1	196.37	196.73	210.35	221.32	148.38	159.5	173.03	173.21	158.16	154.34	120.01	110.23	147.89	143.4	55.98	58.74	77.32	62.75	109.01	103.46	91.15	83.26
Raw Count	comp204328_c0_seq1	2860.18	2770	3443	3622	2127	2357.01	3186	3327.07	2817	2947.21	2184.02	1911	3252	2872.98	1993	1930.69	2553.99	2108	3082	2858	2427	2136

(b)

Figure 2: **PdumBase Expression data tab interface:** **(a)** The upper frame displays mean FPKM and raw counts data, from samples as a pool. **(b)** Lower frame displays expression data from individual replicas.

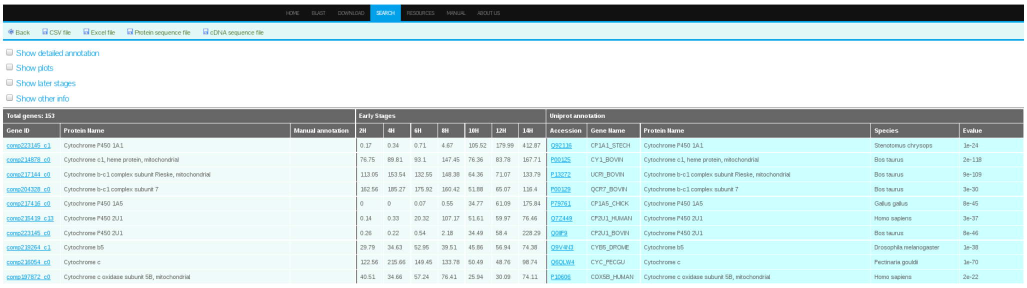

Total genes: 153			Early Stages							Uniprot annotation				
Gene ID	Protein Name	Manual annotation	2H	4H	6H	8H	10H	12H	14H	Accession	Gene Name	Protein Name	Species	Evalue
comp223145_c1	Cytochrome P450 1A1		0.17	0.34	0.71	4.67	105.52	179.99	412.87	Q92116	CP1A1_STECH	Cytochrome P450 1A1	Stenotomus chrysops	1e-24
comp214878_c0	Cytochrome c1, heme protein, mitochondrial		76.75	89.81	93.1	147.45	76.36	83.78	167.71	P00125	CY1_BOVIN	Cytochrome c1, heme protein, mitochondrial	Bos taurus	2e-118
comp217144_c0	Cytochrome b-c1 complex subunit Rieske, mitochondrial		113.05	153.54	132.55	148.38	64.36	71.07	133.79	P13272	UCRI_BOVIN	Cytochrome b-c1 complex subunit Rieske, mitochondrial	Bos taurus	9e-109
comp204328_c0	Cytochrome b-c1 complex subunit 7		162.56	185.27	175.92	160.42	51.88	65.07	116.4	P00129	QCR7_BOVIN	Cytochrome b-c1 complex subunit 7	Bos taurus	3e-30
comp217416_c0	Cytochrome P450 1A5		0	0	0.07	0.55	34.77	61.09	175.84	P79761	CP1A5_CHICK	Cytochrome P450 1A5	Gallus gallus	8e-45
comp215419_c13	Cytochrome P450 2U1		0.14	0.33	20.32	107.17	51.61	59.97	76.46	Q7Z449	CP2U1_HUMAN	Cytochrome P450 2U1	Homo sapiens	3e-37
comp223145_c0	Cytochrome P450 2U1		0.26	0.22	0.54	2.18	34.49	58.4	228.29	Q0IIF9	CP2U1_BOVIN	Cytochrome P450 2U1	Bos taurus	8e-46
comp219264_c1	Cytochrome b5		29.79	34.63	52.95	39.51	45.86	56.94	74.38	Q9V4N3	CYB5_DROME	Cytochrome b5	Drosophila melanogaster	1e-38
comp216054_c0	Cytochrome c		122.56	215.66	149.45	133.78	50.49	48.76	98.74	Q6QLW4	CYC_PECGU	Cytochrome c	Pectinaria gouldii	1e-70
comp197872_c0	Cytochrome c oxidase subunit 5B, mitochondrial		40.51	34.66	57.24	76.41	25.94	30.09	74.11	P10606	COX5B_HUMAN	Cytochrome c oxidase subunit 5B, mitochondrial	Homo sapiens	2e-22

Figure 3: **PdumBase Search results interface** displays Uniprot annotation data on the rightmost panel. Annotation data includes accession number, gene name, protein name, species and E-value. Clicking on the accession number will redirect to the UniProt page for that particular protein.

Uniprot annotation

The **PdumBase** search results interface retrieves the Uniprot annotation data, displaying the Uniprot accession number, gene name, protein name, the species of annotation origin, and the E-value (see Figure 3). The annotation was performed using BLASTP by aligning the transcripts with predicted open reading frames (ORF) against non-redundant SwissProt databases. A total 31,806 transcripts (17,213 genes) retrieved at least one hit using an E-value cutoff of 10^{-10}. Among the annotated transcripts, 26% aligned to human and 19% to mouse proteins.

Pfam Annotation

We also annotated for potential protein domains by aligning all transcripts against the Pfam database. The Pfam annotation can be accessed in the database web interface by selecting the option **"Show detailed annotation"** on the search results page, or by clicking on the tab **"Annotation"** after having selected a particular transcript from the result interface (see Figure 4).
Annotation was performed using HMMER. We were able to assign Pfam domains to 32,464 transcripts (18,146 genes), identifying a total of 431,701 Pfam domains. Furthermore, out of the transcripts with domain annotations, 28,326 (15,690) were also present in the Uniprot BLASTP annotation.

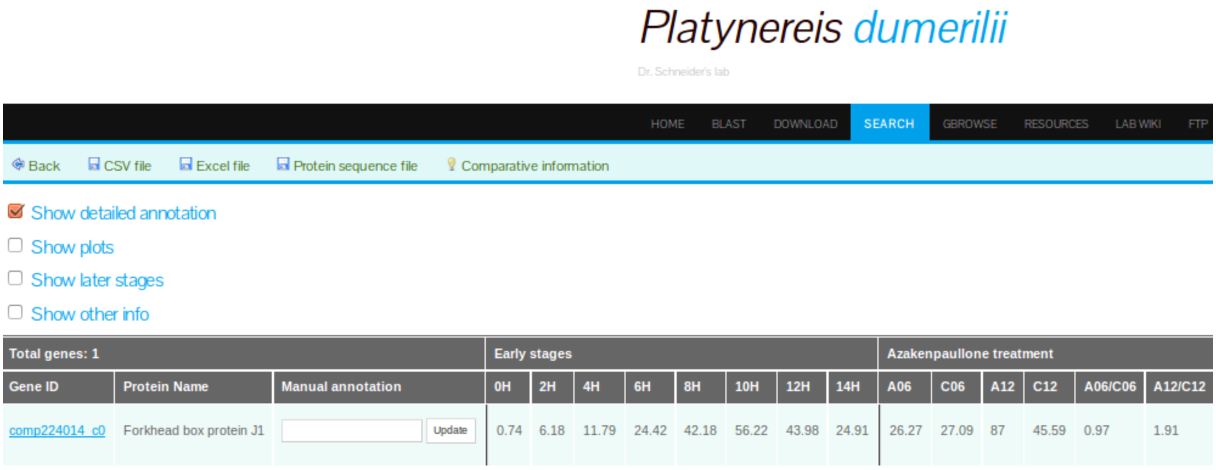

(a)

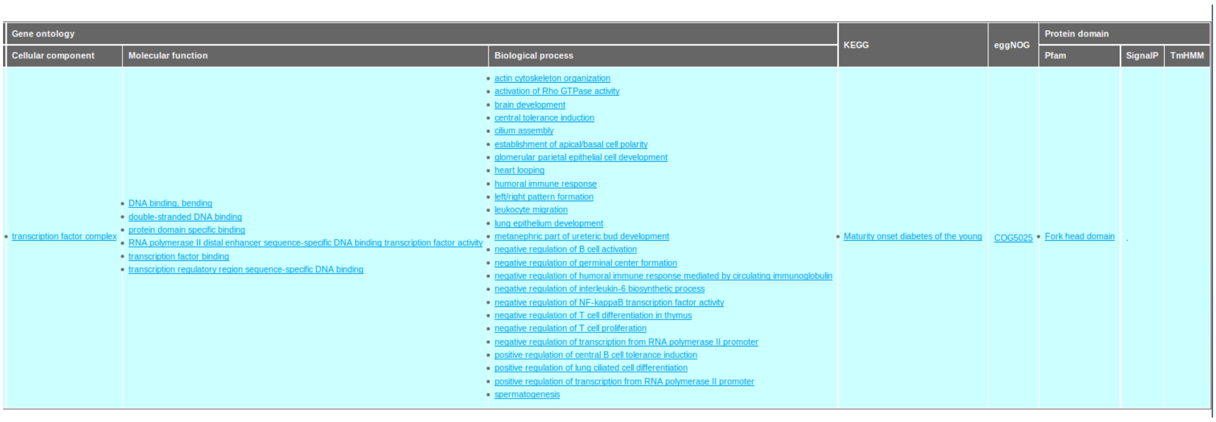

(b)

Figure 4: **PdumBase Search results interface. (a)** The search result page allows to customize the information displayed by checking one or more options from the left top corner. **(b)** Selecting the option: **"Show detailed annotation"** will show detailed gene ontology, KEGG Pathways and protein domain annotation.

KEGG Pathways Annotation

Identifying the active biological pathways in early stages is crucial to decipher the mechanisms involved in the diversification of embryonic cells. The Kyoto Encyclopedia of Genes and Genomes (KEGG) provides well-annotated pathway databases including metabolism, genetic and cellular processing.
Our assembled transcripts were mapped to KEGG pathways. In total, 18,532 transcripts (10,132 genes) are associated with the known KEGG pathways.

In our database, the KEEG annotation is accessible by selecting the option **"Show detailed annotation"** as seen in Figure 4.

Gene Ontology Annotation

The assembled transcripts were also annotated with Gene ontology (GO) terms of homologous genes. A total of 30,287 transcripts (16,498 genes) could be associated with at least one annotated GO term. The GO annotation shows high enrichment in the function associated with transcription and regulation activities in the biological process and molecular functions. The GO terms related to cell differentiation such as “cell transduction”, “cell adhesion”, “cell division” and “cell cycle” are also enriched.

All annotation information for a given transcript is summarized and displayed in the annotation tab interface (see Figure 5).

It is worth pointing out that one important feature of our database is that the search interface allows for the submission of searches by Keyword, Pfam, Gene Ontology, and Kegg Pathway, making it possible to narrow down a request by a particular annotation of interest.

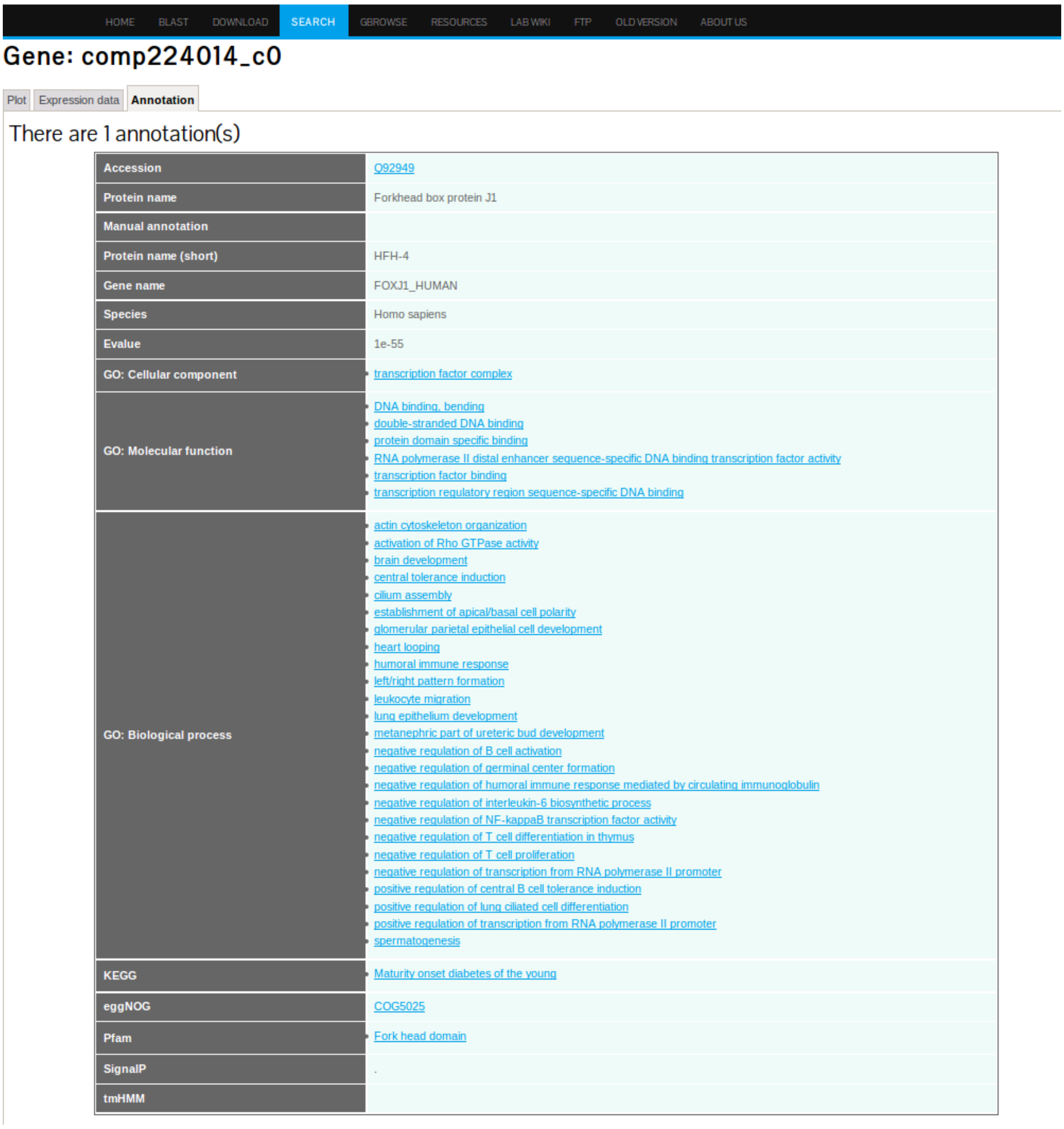
HOME BLAST DOWNLOAD SEARCH GBROWSE RESOURCES LAB WIKI FTP OLD VERSION ABOUT US

Gene: comp224014_c0

Plot | Expression data | Annotation

There are 1 annotation(s)

Accession	Q92949
Protein name	Forkhead box protein J1
Manual annotation	
Protein name (short)	HFH-4
Gene name	FOXJ1_HUMAN
Species	Homo sapiens
Evalue	1e-55
GO: Cellular component	transcription factor complex
GO: Molecular function	DNA binding, bending<br>double-stranded DNA binding<br>protein domain specific binding<br>RNA polymerase II distal enhancer sequence-specific DNA binding transcription factor activity<br>transcription factor binding<br>transcription regulatory region sequence-specific DNA binding
GO: Biological process	actin cytoskeleton organization<br>activation of Rho GTPase activity<br>brain development<br>central tolerance induction<br>cilium assembly<br>establishment of apical/basal cell polarity<br>glomerular parietal epithelial cell development<br>heart looping<br>humoral immune response<br>left/right pattern formation<br>leukocyte migration<br>lung epithelium development<br>metanephric part of ureteric bud development<br>negative regulation of B cell activation<br>negative regulation of germinal center formation<br>negative regulation of humoral immune response mediated by circulating immunoglobulin<br>negative regulation of interleukin-6 biosynthetic process<br>negative regulation of NF-kappaB transcription factor activity<br>negative regulation of T cell differentiation in thymus<br>negative regulation of T cell proliferation<br>negative regulation of transcription from RNA polymerase II promoter<br>positive regulation of central B cell tolerance induction<br>positive regulation of lung ciliated cell differentiation<br>positive regulation of transcription from RNA polymerase II promoter<br>spermatogenesis
KEGG	Maturity onset diabetes of the young
eggNOG	COG5025
Pfam	Fork head domain
SignalP	.
tmHMM	

Figure 5: **PdumBase Annotation tab interface.** This tab is available once an entry from the result page has been selected. It is accessible via result page → clicking on a gene or transcript of interest → clicking on the Annotation tab

4. Gene Expression Profiling

Our *Platynereis dumerilii* database: **PdumBase**, includes a detailed gene expression profiling of the early developmental stages (2 to 14hpf). An expression profile can be interpreted as the changes in the abundance of a transcript over time.

Plots depicting these fluctuations of transcript abundance (FPKM) are shown for each transcript. Accessing this data is available via the option **"Show Plots"** on the search result page (Figure 6), or by clicking on the transcript of interest and selecting the tab labeled **"Plots"** (Figure 7). For the purpose of the expression profiling analysis we filtered out low expression transcripts.

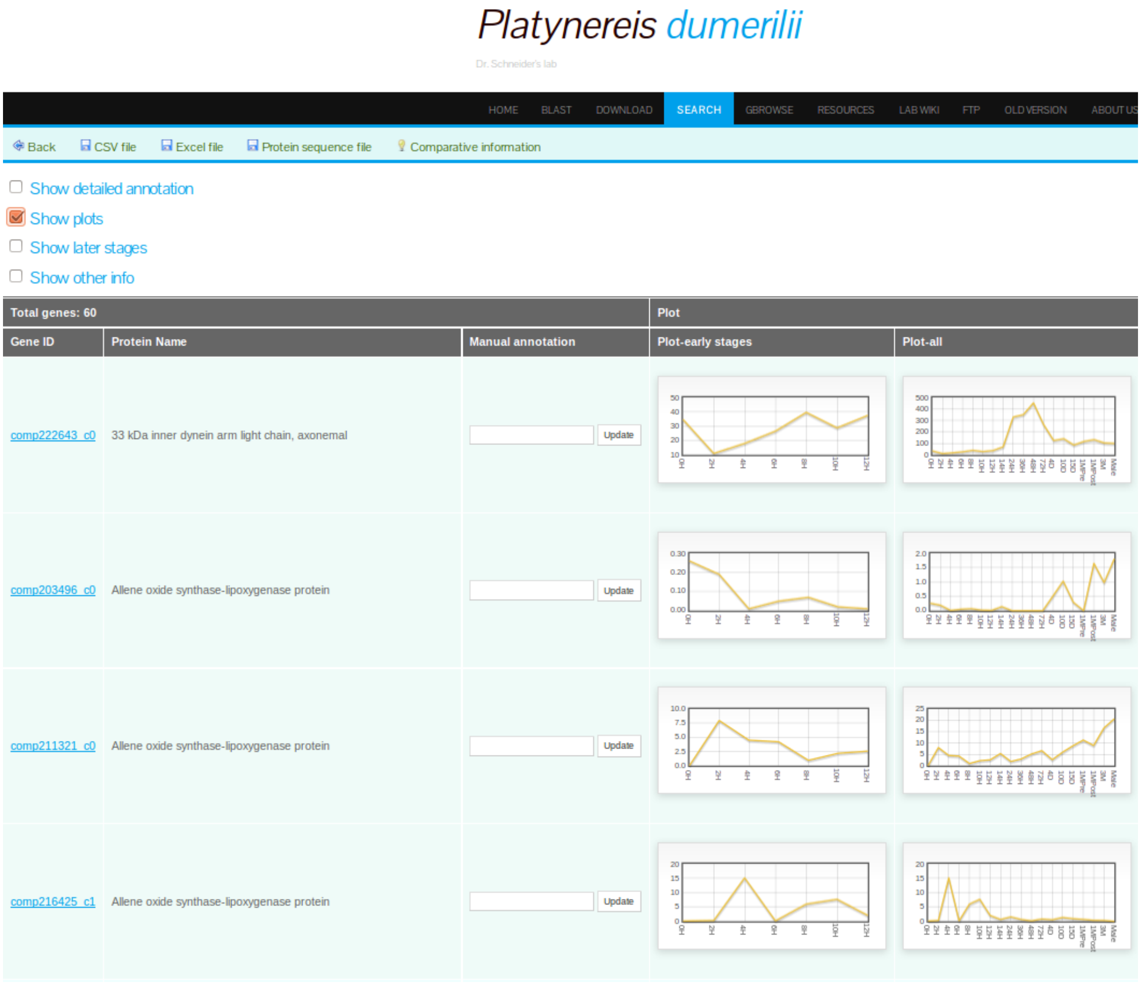

Figure 6: **PdumBase Search result interface.** Expression profile plots are displayed when the option **"Show plots"** is selected.

Among the assembled transcripts with predicted ORF, 18,940 transcripts and 13,160 genes were found to be expressed in at least one of the 7 stages. After clustering the genes according to their expression profile, we found a total of 15 distinct clusters (see Figure 8).

Clusters 1-4 show the obvious maternal signature with a total of 4,302 genes belonging this group. The clusters 10-15 (5827 genes) correspond to the zygotic genes with slightly different activation time points. The clusters 3 and 11 are the major maternal and zygotic groups respectively showing slow decreased and increased expression patterns. The 6th, 7th, and 8th cluster contain a set of genes whose RNAs were mainly expressed at 4, 6 and 8 hours and degraded after these stages. The 9th cluster is a less dynamic group, showing stable expression throughout all stages.

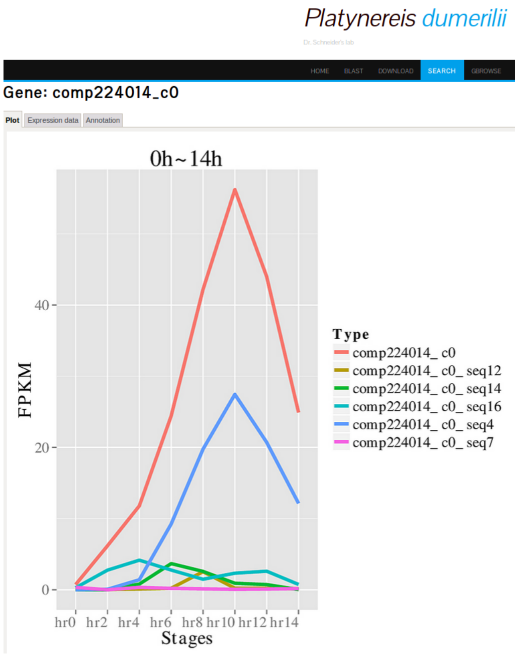

Figure 7: **PdumBase Plot tab interface**. Shows the expression profile plot for a given transcript.

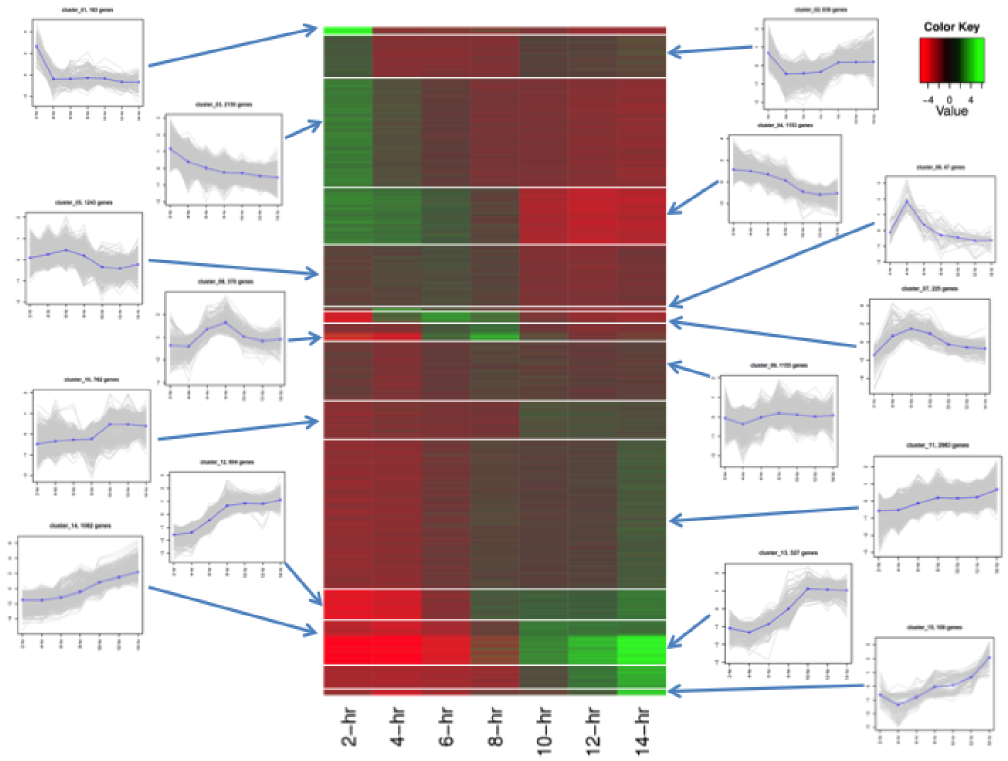

Figure 8: Heat map of 13,160 expressed genes clustered into 15 groups according to the time series patterns.

Access to the cluster information is available by selecting the option **”Show other info”** on the result search page and then, for a selected transcript/gene, clicking on the icon under **”Coexpression info”**. The first tab of the new results page will display all the genes in the same cluster, along with other expression data (see Figure 9).

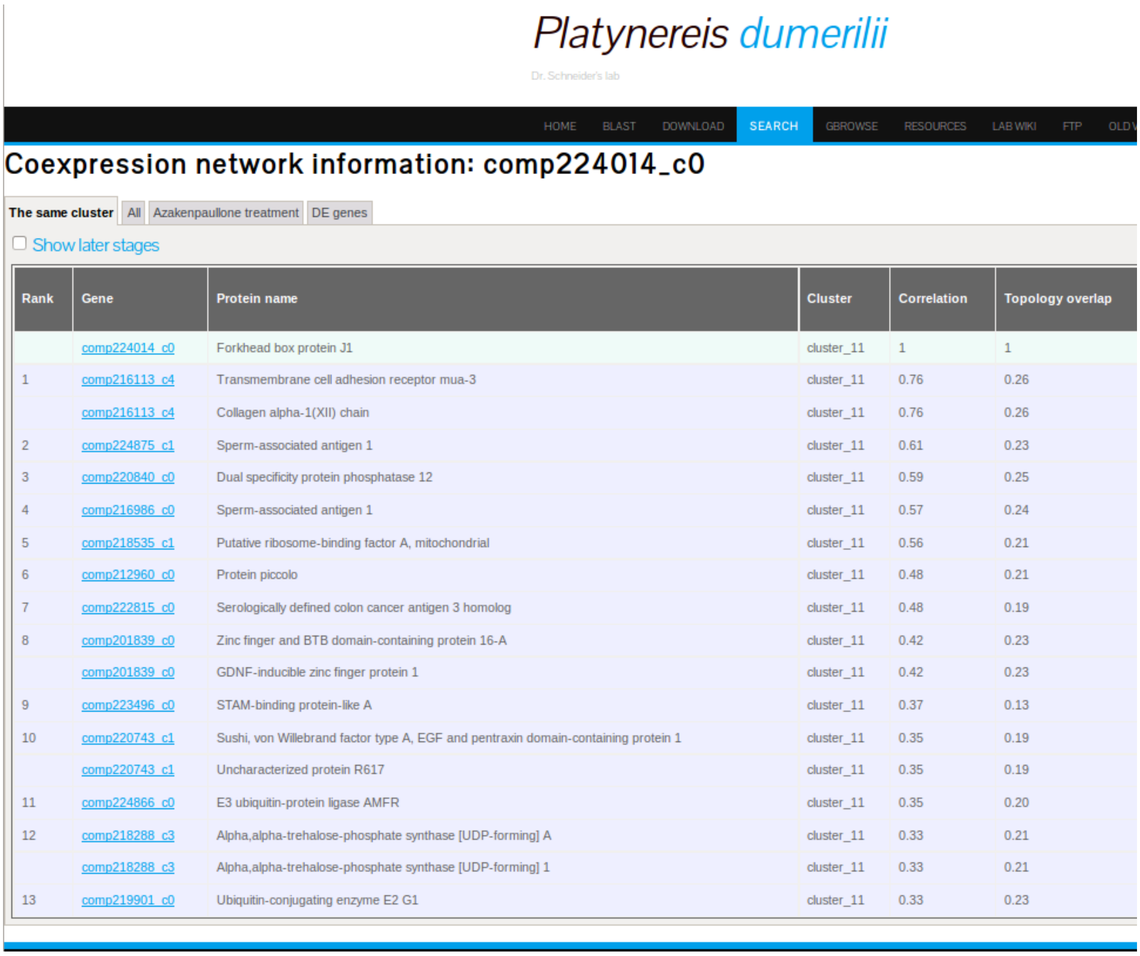
Platynereis dumerilii
Dr. Schneider's lab
HOME BLAST DOWNLOAD SEARCH GBROWSE RESOURCES LAB WIKI FTP OLD V

Coexpression network information: comp224014_c0

The same cluster | All | Azakenpaullone treatment | DE genes

Show later stages

Rank	Gene	Protein name	Cluster	Correlation	Topology overlap
	comp224014_c0	Forkhead box protein J1	cluster_11	1	1
1	comp216113_c4	Transmembrane cell adhesion receptor mua-3	cluster_11	0.76	0.26
	comp216113_c4	Collagen alpha-1(XII) chain	cluster_11	0.76	0.26
2	comp224875_c1	Sperm-associated antigen 1	cluster_11	0.61	0.23
3	comp220840_c0	Dual specificity protein phosphatase 12	cluster_11	0.59	0.25
4	comp216986_c0	Sperm-associated antigen 1	cluster_11	0.57	0.24
5	comp218535_c1	Putative ribosome-binding factor A, mitochondrial	cluster_11	0.56	0.21
6	comp212960_c0	Protein piccolo	cluster_11	0.48	0.21
7	comp222815_c0	Serologically defined colon cancer antigen 3 homolog	cluster_11	0.48	0.19
8	comp201839_c0	Zinc finger and BTB domain-containing protein 16-A	cluster_11	0.42	0.23
	comp201839_c0	GDNF-inducible zinc finger protein 1	cluster_11	0.42	0.23
9	comp223496_c0	STAM-binding protein-like A	cluster_11	0.37	0.13
10	comp220743_c1	Sushi, von Willebrand factor type A, EGF and pentraxin domain-containing protein 1	cluster_11	0.35	0.19
	comp220743_c1	Uncharacterized protein R617	cluster_11	0.35	0.19
11	comp224866_c0	E3 ubiquitin-protein ligase AMFR	cluster_11	0.35	0.20
12	comp218288_c3	Alpha,alpha-trehalose-phosphate synthase [UDP-forming] A	cluster_11	0.33	0.21
	comp218288_c3	Alpha,alpha-trehalose-phosphate synthase [UDP-forming] 1	cluster_11	0.33	0.21
13	comp219901_c0	Ubiquitin-conjugating enzyme E2 G1	cluster_11	0.33	0.23

Figure 9: **PdumBase Coexpression information interface**. Displays all the transcripts/genes in the same cluster of a given component, shows protein name, correlation and topology overlap.

5. Coexpression Networks

A coexpression network is a correlation network that describes the pairwise correlation patterns of expression data. When a set of genes are highly correlated, they may share similar biological function or be involved in the same biological pathway. A coexpression network can also be used for identifying hub genes which have high connectivity to other genes in a cluster. We used weighted correlation network analysis (WGCNA) to analyze *Platynereis dumerilii* expression profiling data.

For this analysis, we included a total of 13,192 genes whose FPKM was ≥ 1 for at least one sample. Correlation values and topology overlap for the coexpression networks can be found in the database on the **Coexpression information interface**. This page can be reached from the search result interface by selecting the option **"Show other info"** and by clicking on the icon under the column **"Coexpression info"** in the results table. The **Coexpression information interface** is shown in Figure 9.

6. Comparative Transcriptome Data

Ortholog Expression

With the aim of identifying conserved stages of development, we gathered publicly available expression data from five species for which we then identified orthologs w.r.t. *Platynereis dumerilii* (see Tables 3 and 4) and proceeded to establish global comparison expression profiles among the ortholog groups.

The ortholog expression data for a particular *Platinereis dumerilii* transcript, can be found in our database by selecting the option **"Show other info"** and by clicking on the icon under the column *Ortholog Expressions* for the specific transcript of interest. The resulting interface will display the ID number and expression data for the orthologs found for that transcript/gene in the other 5 species (see Figure 10).

Platynereis_dumerilii

SS00	SS02	SS04	SS06	SS08	SS10	SS12	SS14	H24	H36	H48	H72	D4	D10	D15	M1Pre	M1Post	M3	Male	Female	A06	C06	A12	C12	A06/C06	A12/C12	Protein_name
2.4	58.2	17.94	16.65	16.62	13.94	9.99	8.96	9.54	18.04	13.48	17.15	15.95	15.55	12.92	12.4	11.64	8.34	9.33	32.08	17.74	20.17	17	12.54	0.88	1.36	Transcription factor RFX3

Ascaris_suum

Gene_id	oocyte	zygote1	zygote2	zygote3	zygote4	24h	46h	64h	96h	116h	7D	L1	L2	Protein_name
ASU_01592	0.5	1.1	2	4.4	6	9	8.4	8.4	7.1	13.1	59.3	20.5	12.6	Abnormal dauer formation protein 19

Danio_rerio

Gene_id	2_cell	64_cell	3.5hpf	6hpf	9hpf	Protein_name
ENSDARG00000014550	3.5353	3.97681	5.66213	2.09896	1.26579	Transcription factor RFX3

Xenopus_tropicalis

Gene_id	cell2	cell4	cell8	cell16	Stage6	Stage8	Stage9	Stage10	Stage11_12	Stage13_14	Stage15	Stage16_18	Stage19	Stage20_21	Stage22_23	Stage24_26	Stage28	Stage31_32
ENSXETG00000005567	17.2043	19.0805	21.6319	18.7191	18.3343	18.1596	17.5738	12.3135	6.93579	11.2533	16.3181	17.9518	15.4797	11.0376	11.0063	10.407	9.75551	13.9335

Homo_sapiens

Gene_id	HUES64	Endoderm	Ectoderm	Mesoderm	Hepatoblast_like	Protein_name
RFX1	5.42569	5.0263	6.21082	5.92104	12.4656	

Nematostella_vectensis

Gene_id	2h	7h	12h	24h	5d	10d	Protein_name
e_gw.43.110.1	166.029	115.501	134.056	73.32	50.1426	43.2087	Regulatory factor X 3

Strongylocentrotus_purpuratus

Gene_id	0h	10h	18h	24h	30h	40h	48h	56h	64h	72h	Protein_name
WHL22.19679	89.0913	83.5982	69.7351	28.2143	27.0699	30.8884	38.166	33.884	36.9231	44.9459	Regulatory factor X 3

© SQS group, GDCB, Iowa State University by Hsien-chao Chou

Figure 10: **PdumBase Ortholog expression profile interface**. Displays the expression data from the selected *Platynereis dumerilii* gene and the orthologs genes found in the other species along with their expression and annotation data (when available).

Table 3: Species and number of protein sequences for comparative analysis

Species	Number of sequences
Platynereis dumerilii	28,580
Danio rerio	26,241
Xenopus tropicales	18,442
Homo sapiens	23,393
Nematostella vectensis	27,273
Ascaris suum	15,446

Table 4: Number of orthologs genes between the 6 species

Species	*Platynereis dumerilii*	*Danio rerio*	*Xenopus tropicalis*	*Homo sapiens*	*Nematostella vectensis*	*Ascaris suum*
Platynereis dumerilii		5635	5402	5051	5840	3654
Danio rerio			10784	10246	6731	4307
Xenopus tropicales				10284	6415	4140
Homo sapiens					6094	3941
Nematostella vectensis						4245
Ascaris suum						

Ortholog Groups

We also identified orthologs genes for 18 selected species (Table 5) using the program OrthoMCL. This program runs all versus all Blastp queries among all the protein sequences from these 18 species and selects the best reciprocal blast hits. Once the orthologs genes were identified, phylogenetic trees were assembled using RaxMl.

Table 5: Species and number of genes used to find orthologs groups

Class	Code	Species	Number of genes
Lophotrochozoa	pdu	*Platynereis dumerilii*	28,580
Lophotrochozoa	cte	*Capitella teleta*	32,415
Lophotrochozoa	hro	*Helobdella robusta*	23,423
Lophotrochozoa	lgi	*Lottia gigantea*	23,851
Lophotrochozoa	cgi	*Crassostrea gigas*	26,089
Ecdysozoa	dpu	*Daphnia pulex*	30,907
Ecdysozoa	tca	*Tribolium castaneum*	16,524
Ecdysozoa	dme	*Drosophila melanogaster*	13,937
Deuterostomia	spu	*Strongylocentrotus purpuratus*	20,759
Deuterostomia	sko	*Saccoglossus kowalevskii*	34,239
Deuterostomia	bfo	*Branchiostoma floridae*	50,817
Deuterostomia	dre	*Danio rerio*	26,459
Deuterostomia	xtr	*Xenopus tropicalis*	18,442
Deuterostomia	hsa	*Homo sapiens*	23,393
Prebilateria	nve	*Nematostella vectensis*	27,273
Prebilateria	aqu	*Amphimedon queenslandica*	29,883
Prebilateria	tad	*Trichoplax adhaerens*	11,520
Preanimalia	mbr	*Monosiga brevicollis*	9,196

To access the ortholog genes for a given *Platynereis dumerilii* transcript/gene, select the option **"show other info"**. If ortholog groups are found for that particular transcript, a check-mark will appear under the field **"Ortholog groups"**. Clicking on this icon will open a new interface with four tabs: **"List"**, **"Tree-ML"**, **"Tree-Parsimony"**, and **Alignment** (see Figures 11, 12, and 13 respectively).

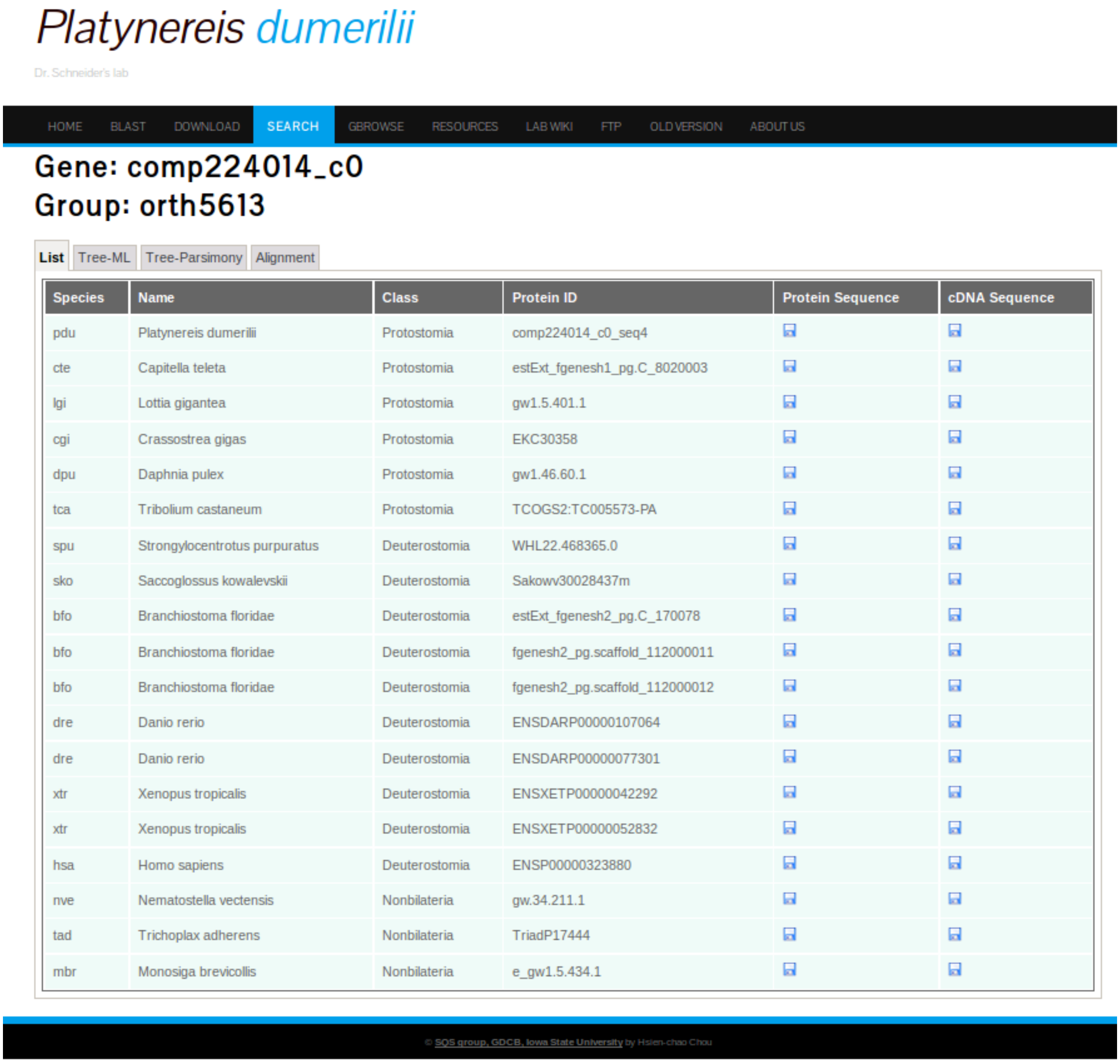

Species	Name	Class	Protein ID	Protein Sequence	cDNA Sequence
pdu	Platynereis dumerilii	Protostomia	comp224014_c0_seq4		
cte	Capitella teleta	Protostomia	estExt_fgenesh1_pg.C_8020003		
lgi	Lottia gigantea	Protostomia	gw1.5.401.1		
cgi	Crassostrea gigas	Protostomia	EKC30358		
dpu	Daphnia pulex	Protostomia	gw1.46.60.1		
tca	Tribolium castaneum	Protostomia	TCOGS2:TC005573-PA		
spu	Strongylocentrotus purpuratus	Deuterostomia	WHL22.468365.0		
sko	Saccoglossus kowalevskii	Deuterostomia	Sakowv30028437m		
bfo	Branchiostoma floridae	Deuterostomia	estExt_fgenesh2_pg.C_170078		
bfo	Branchiostoma floridae	Deuterostomia	fgenesh2_pg.scaffold_112000011		
bfo	Branchiostoma floridae	Deuterostomia	fgenesh2_pg.scaffold_112000012		
dre	Danio rerio	Deuterostomia	ENSDARP00000107064		
dre	Danio rerio	Deuterostomia	ENSDARP00000077301		
xtr	Xenopus tropicalis	Deuterostomia	ENSXETP00000042292		
xtr	Xenopus tropicalis	Deuterostomia	ENSXETP00000052832		
hsa	Homo sapiens	Deuterostomia	ENSP00000323880		
nve	Nematostella vectensis	Nonbilateria	gw.34.211.1		
tad	Trichoplax adherens	Nonbilateria	TriadP17444		
mbr	Monosiga brevicollis	Nonbilateria	e_gw1.5.434.1		

Figure 11: **PdumBase List tab interface** under Ortholog groups. Shows the species list, code, name, ortholog protein ID and contains links to access/download the protein and cDNA sequences in Fasta format.

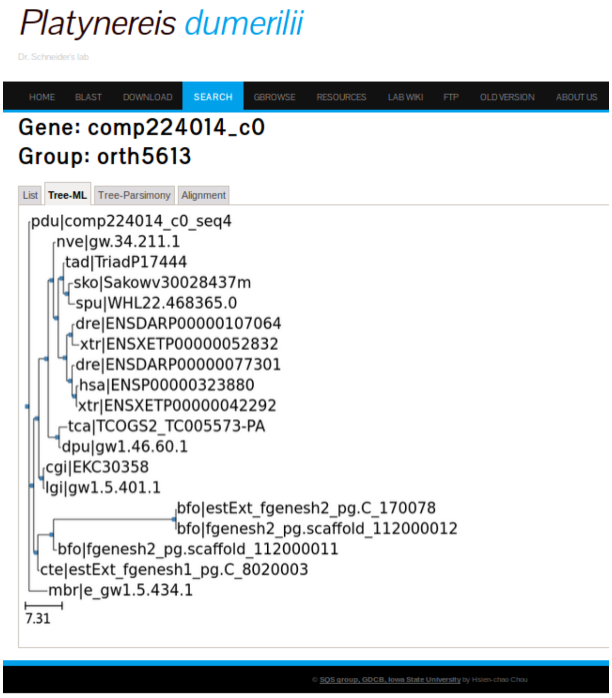

(a)

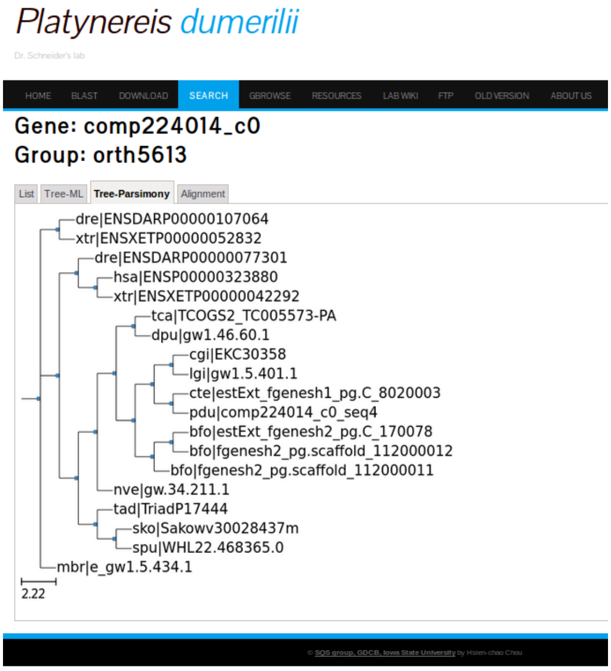

(b)

Figure 12: **PdumBase Ortholog groups interface: (a)** Phylogenetic tree among ortholog genes displayed under **Tree-ML tab (b)** Phylogenetic tree displayed under **Tree-Parsimony tab**. Both trees show the species code and the transcript/gene ID.

```
mbr|e_gw1.5.434.1                LIFMAMQQADKPKLALSEIYDFIVNNFAWYRMADPGWKNSIRHNLSQEKA
xtr|ENSXETP00000052832           LICMAMEASQQRKLTLSAIYSWITQNFCYYRHADPSWQNSIRHNLSLNKC
dre|ENSDARP00000107064           LICMAMQASNKTKITLSAIYSWITENFCYYRYAEPSWQNSIRHNLSLNKC
nve|gw.34.211.1                  LICMAMRDTKRVKITLSAIYKWIKENFMFYRVADPTWQNSIRHNLSLNKC
xtr|ENSXETP00000042292           LICMAMQASKKTKITLSAIYKWITDNFCYFRHADPTWQNSIRHNLSLNKC
hsa|ENSP00000323880              LICMAMQASKATKITLSAIYKWITDNFCYFRHADPTWQNSIRHNLSLNKC
dre|ENSDARP00000077301           LICMAMQASKKTKITLSCIYKWITDNFCYFRHADPTWQNSIRHNLSLNKC
dpu|gw1.46.60.1                  LICMAMKS-NKHKMTLSSIYKWIKENFLYYRNVDPSWQNSIRHNLSLNKC
tca|TCOGS2_TC005573-PA           LICMAMGK-NGNKMTLSAIYHWIRENFLYYRKAHPSWQNSIRHNLSLNKC
spu|WHL22.468365.0               LIWMAMKESKKHKITLSSIYKWITENFKYYQVADPSWQNSIRHNLSLNKC
tad|TriadP17444                  LICMAMKESKKSKITLSAIYNWIRENFMYYRIADPSWQNSIRHNLSLNKC
lgi|gw1.5.401.1                  LIGMAMKETQKQKITLSAIYNWITDNFMYYRMADPSWQNSIRHNLSLNKC
pdu|comp224014_c0_seq4           LICMAMKETNRNKITLSGIYSWITENYMYYRVADPSWQNSIRHNLSLNKC
cte|estExt_fgenesh1_pg.C_80200   LICMAMKESKKNKVTLSGIYNWITENFMYYRMADPSWQNSIRHNLSLNKC
cgi|EKC30358                     ---MAMKETKKSKITLSAIYNWITDNFMYYRLADPSWQNSIRHNLSLNKC
sko|Sakowv30028437m              LICMAMKETKKNKITLSAIYKWIQDNFMYYKVAEPSWQNSIRHNLSLNKC
bfo|estExt_fgenesh2_pg.C_17007   IDQVLSEPSHLNESTSNILNTNLPPSPPSSEGYRHPWEEASELDSIVDMN
bfo|fgenesh2_pg.scaffold_11200   IDQVLSEPSHLNESTSNILNTNLPPSPPSSEGYRHPWEEASELDSIVDMN
bfo|fgenesh2_pg.scaffold_11200   LICMAMKETKKSKITLSDIYKWIKTNFKYYEMAEPSWQISRQLYSSNKR-
                                    :     .  : : . :   :  .    .     *: :  .      .
```

Figure 13: **PdumBase Alignment tab interface** under Ortholog groups. Displays CLUSTAL 2.1 multiple sequence alignment.

Tutorial Example: Searching By Keyword

This section will show some of the *Platynereis dumerilii* web database features through exemplary searches using the blast info search function.

Search

The search interface allows to submit searches under different criteria: By Keyword, Pfam, SingalIP, TmHMM, EggNog, Gene Ontology, and KEEG Pathway (Figure 14). By searching under different or combined fields, the search can be customized according to the user needs.

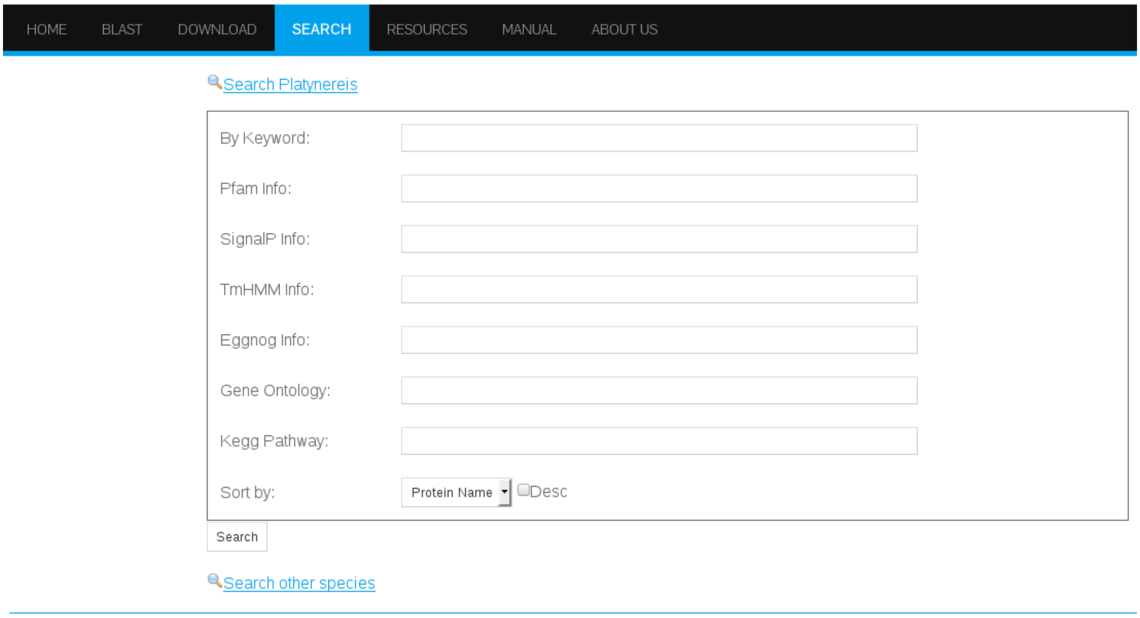

Figure 14: **PdumBase Search interface**.

In addition, the search interface offers the option of selecting a sorting criteria to retrieve the results according to the expression values from any stage (0 to 14hpf) (Figure 15). This feature can be particularly convenient when searching with terms that might result in a multitude of hits such as "cell cycle" which retrieves more than 1000 genes, or "membrane" with around 500 hits. Therefore, searching for general terms might result in a request which could take more than 60 seconds to load. Please allow time for those general searches to load.

On the other hand, when searching for a particular gene name, for instance the transcription factor **FoxA2** in the field Blast Info, the most likely outcome will be one single hit displaying the *Platynereis dumerilii* transcript/gene with that particular annotation.

Search Results

The resulting search results interface displays by default the transcript or gene model ID, protein name, expression data as mean FPKM from early stages (0 to 14 hpf), expression data in inhibitor experiment, and annotation information (Figure 16).

In addition, the results interface allows to expand the results displayed by selecting from the options on the left upper corner. The user can select one or more options according to his/her particular research needs (see also Expanded search result options Section).

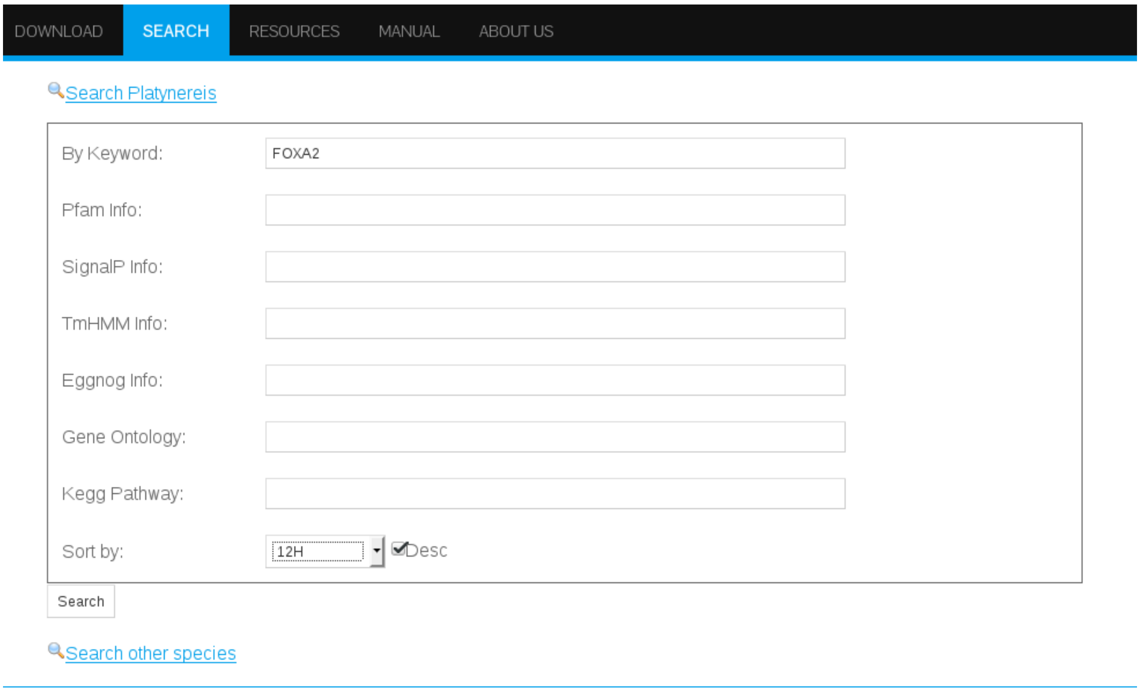

Figure 15: **PdumBase Search interface**. Searching for FoxA2

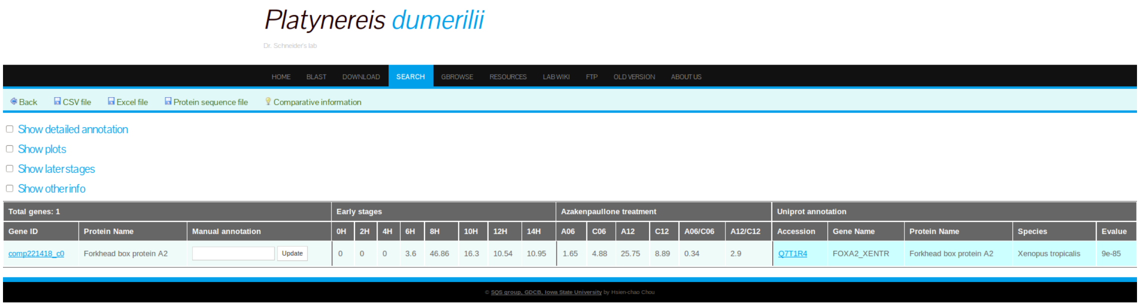

Figure 16: **PdumBase Search result interface**. shows Gene ID, expression data from early stages and from inhibitor experiment, and annotation information. The data retrieve options are found on the left upper corner.

Access to Detailed Information

Clicking on the gene model for FoxA2 ”comp221418_co” will give access to the detailed data results interface. The detailed data result page has three tabs: Plot, Expression data and Annotation, from which different information can be accessed.

The Plot Tab

Clicking on the Plot tab will display expression profile data (FPKM values against stages) for early and late states (Figure 17).

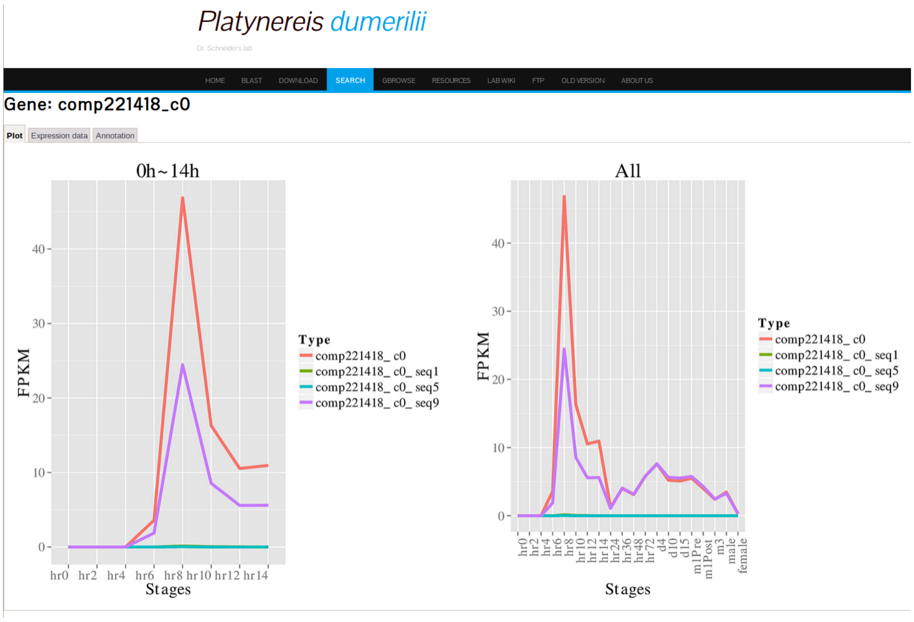

Figure 17: **PdumBase Plot tab** from Detailed data results interface. Displaying expression profile plots for FoxA2.

The Expression Data Tab

The Expression data tab will show mean and individual sample FPKM values as well as raw counts (Figure 18).

The Annotation Tab

Clicking the annotation tab will retrieve a summary of all annotation related information including: Species from which the annotation was obtained, GO extended annotation, KEEG pathways, EggNog, and Pfam domains (see figure 19).

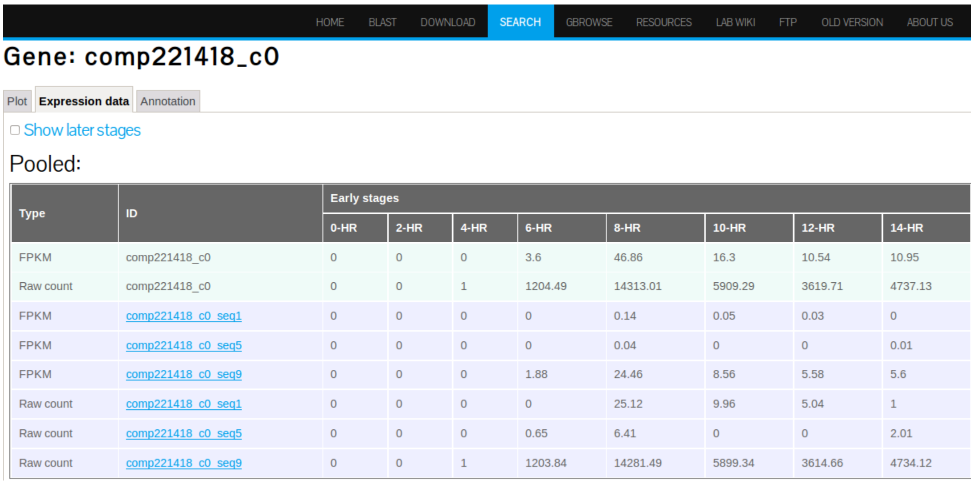

HOME BLAST DOWNLOAD SEARCH GBROWSE RESOURCES LAB WIKI FTP OLD VERSION ABOUT US

Gene: comp221418_c0

Plot | Expression data | Annotation

Show later stages

Pooled:

Type	ID	Early stages							
		0-HR	2-HR	4-HR	6-HR	8-HR	10-HR	12-HR	14-HR
FPKM	comp221418_c0	0	0	0	3.6	46.86	16.3	10.54	10.95
Raw count	comp221418_c0	0	0	1	1204.49	14313.01	5909.29	3619.71	4737.13
FPKM	comp221418_c0_seq1	0	0	0	0	0.14	0.05	0.03	0
FPKM	comp221418_c0_seq5	0	0	0	0	0.04	0	0	0.01
FPKM	comp221418_c0_seq9	0	0	0	1.88	24.46	8.56	5.58	5.6
Raw count	comp221418_c0_seq1	0	0	0	0	25.12	9.96	5.04	1
Raw count	comp221418_c0_seq5	0	0	0	0.65	6.41	0	0	2.01
Raw count	comp221418_c0_seq9	0	0	1	1203.84	14281.49	5899.34	3614.66	4734.12

(a)

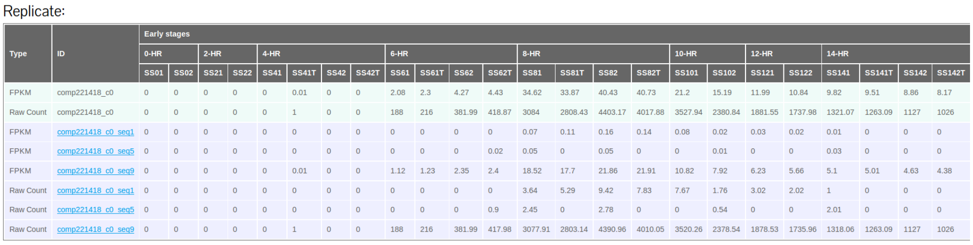

Replicate:

Type	ID	Early stages											
		0-HR		2-HR		4-HR				6-HR			
		SS01	SS02	SS21	SS22	SS41	SS41T	SS42	SS42T	SS61	SS61T	SS62	SS62T
FPKM	comp221418_c0	0	0	0	0	0	0.01	0	0	2.08	2.3	4.27	4.43
Raw Count	comp221418_c0	0	0	0	0	0	1	0	0	188	216	381.99	418.87
FPKM	comp221418_c0_seq1	0	0	0	0	0	0	0	0	0	0	0	0
FPKM	comp221418_c0_seq5	0	0	0	0	0	0	0	0	0	0	0	0.02
FPKM	comp221418_c0_seq9	0	0	0	0	0	0.01	0	0	1.12	1.23	2.35	2.4
Raw Count	comp221418_c0_seq1	0	0	0	0	0	0	0	0	0	0	0	0
Raw Count	comp221418_c0_seq5	0	0	0	0	0	0	0	0	0	0	0	0.9
Raw Count	comp221418_c0_seq9	0	0	0	0	0	1	0	0	188	216	381.99	417.98

Type	ID	8-HR				10-HR		12-HR		14-HR			
		SS81	SS81T	SS82	SS82T	SS101	SS102	SS121	SS122	SS141	SS141T	SS142	SS142T
FPKM	comp221418_c0	34.62	33.87	40.43	40.73	21.2	15.19	11.99	10.84	9.82	9.51	8.86	8.17
Raw Count	comp221418_c0	3084	2808.43	4403.17	4017.88	3527.94	2380.84	1881.55	1737.98	1321.07	1263.09	1127	1026
FPKM	comp221418_c0_seq1	0.07	0.11	0.16	0.14	0.08	0.02	0.03	0.02	0.01	0	0	0
FPKM	comp221418_c0_seq5	0.05	0	0.05	0	0	0.01	0	0	0.03	0	0	0
FPKM	comp221418_c0_seq9	18.52	17.7	21.86	21.91	10.82	7.92	6.23	5.66	5.1	5.01	4.63	4.38
Raw Count	comp221418_c0_seq1	3.64	5.29	9.42	7.83	7.67	1.76	3.02	2.02	1	0	0	0
Raw Count	comp221418_c0_seq5	2.45	0	2.78	0	0	0.54	0	0	2.01	0	0	0
Raw Count	comp221418_c0_seq9	3077.91	2803.14	4390.96	4010.05	3520.26	2378.54	1878.53	1735.96	1318.06	1263.09	1127	1026

(b)

Figure 18: **PdumBase Expression data tab** from Detailed data results interface. **(a)** Displays expression data (mean FPKM and raw counts) from pooled samples from early stages of development. **(d)** show individual replicates expression data for early stages

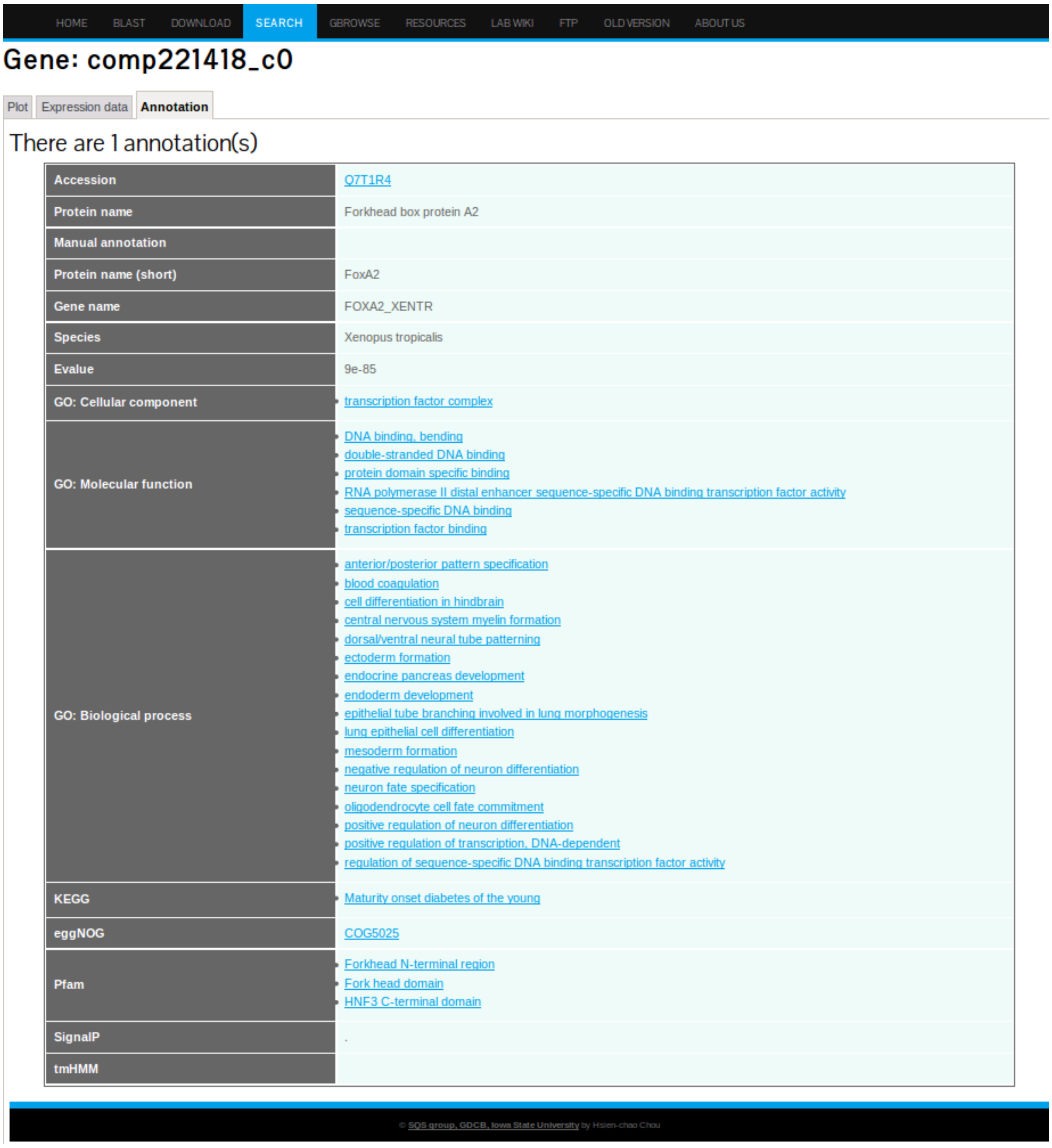

19

Figure 19: **PdumBase Annotation tab** from Detailed data results interface. Displaying detailed annotation information for FoxA2.

Expanded Search Result Options

The search result default data output can be expanded by selecting the options provided in the search results interface (Figure 20).

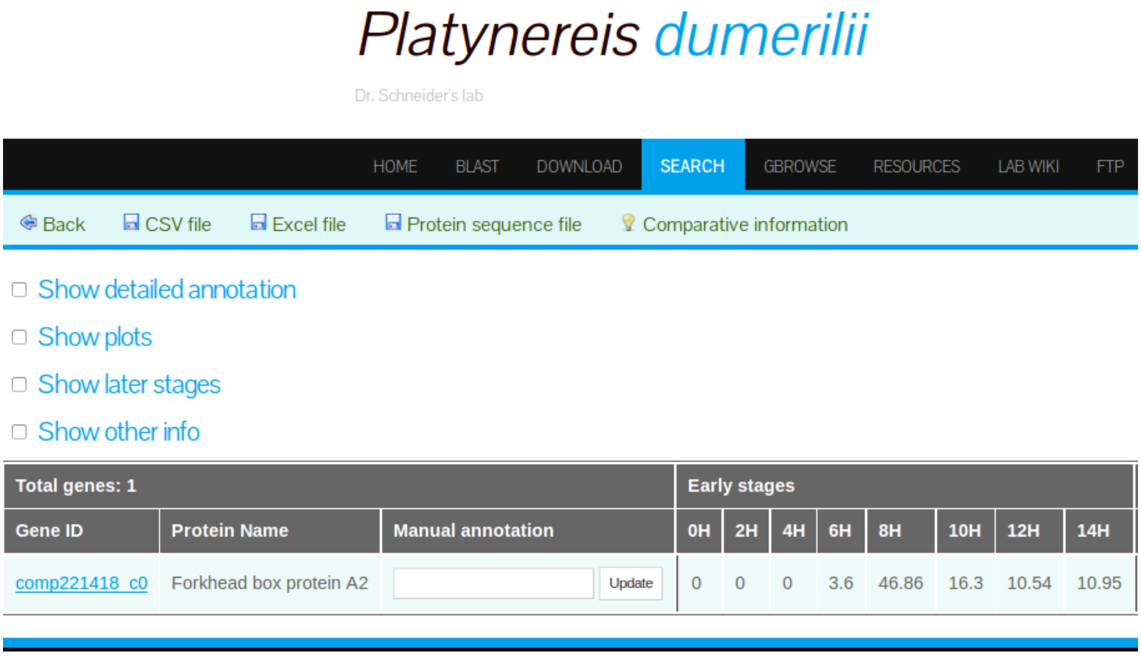

Figure 20: **PdumBase Search results interface** checking the boxes from the search result options on the left will expand the results displayed.

Selecting "Show plots"

Selecting the "show plots" option will retrieve a visual representation of early and late stage expression profile for all the Gene IDs displayed in the search result interface (Figure 21).

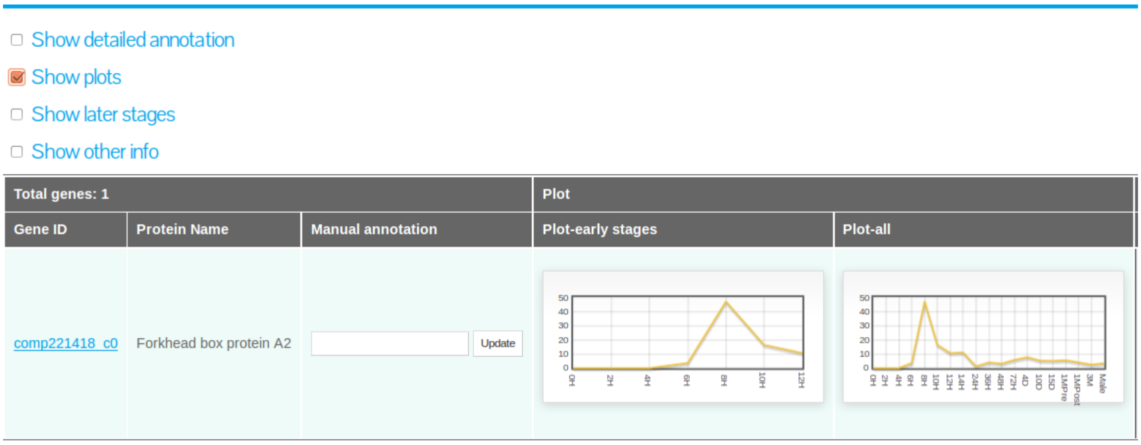

Figure 21: **PdumBase Search results interface** with the option "Show Plots" selected. Expression plots for both, early and late stages are shown for the gene under search: FoxA2.

Selecting "Show later stages"

To display the mean expression data (FPKM) from later stages of development (24hpf to 3M) it is required to select the option "show later stages" as shown in Figure 22.

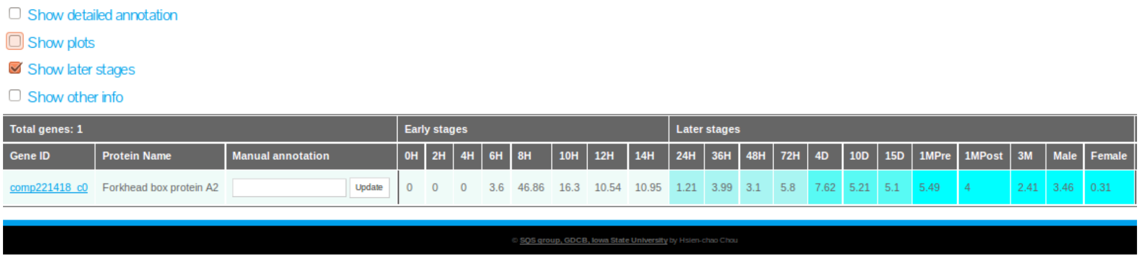

Figure 22: **PdumBase Search results interface** with the option "Show later stages" selected. Here the later stages expression data from FOXA2 is displayed.

Selecting "Show other info"

Clicking "Show other info" provides access to additional data on comparative transcriptomics (see Figure 23):

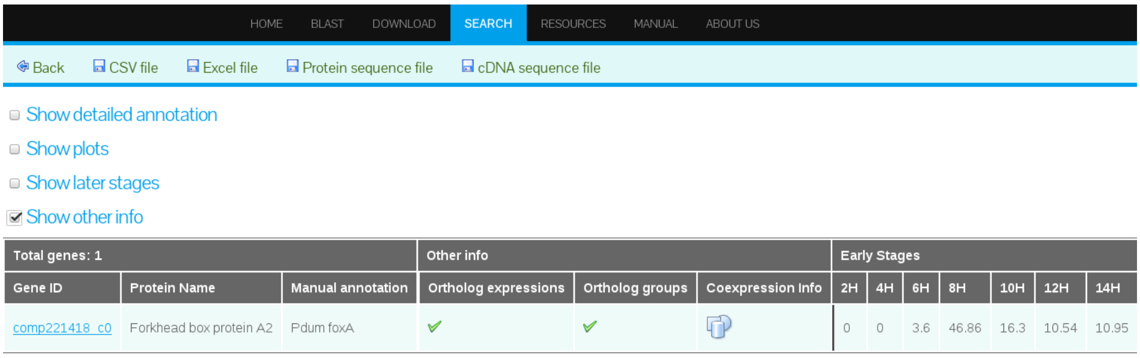

Figure 23: **PdumBase Search results interface** "Show other info" option selected. Additional information links are displayed.

- Ortholog Expressions - if available a green check-mark icon will be displayed.
- Ortholog groups - if available a green check-mark icon will be displayed.
- Coexpression info - if available a blue icon will be displayed.

It is important to mention that the additional data is not available to all the gene models but only to those transcripts for which orthologs genes were identified. See Table 4 for the estimated numbers of orthologs found.

Coexpression link

Selecting the coexpression link gives access to data about the expression profiling and coexpression. ”The same cluster tab” from this interface displays the Gene ID of all genes belonging to the cluster of the gene under search (see Figure 24).

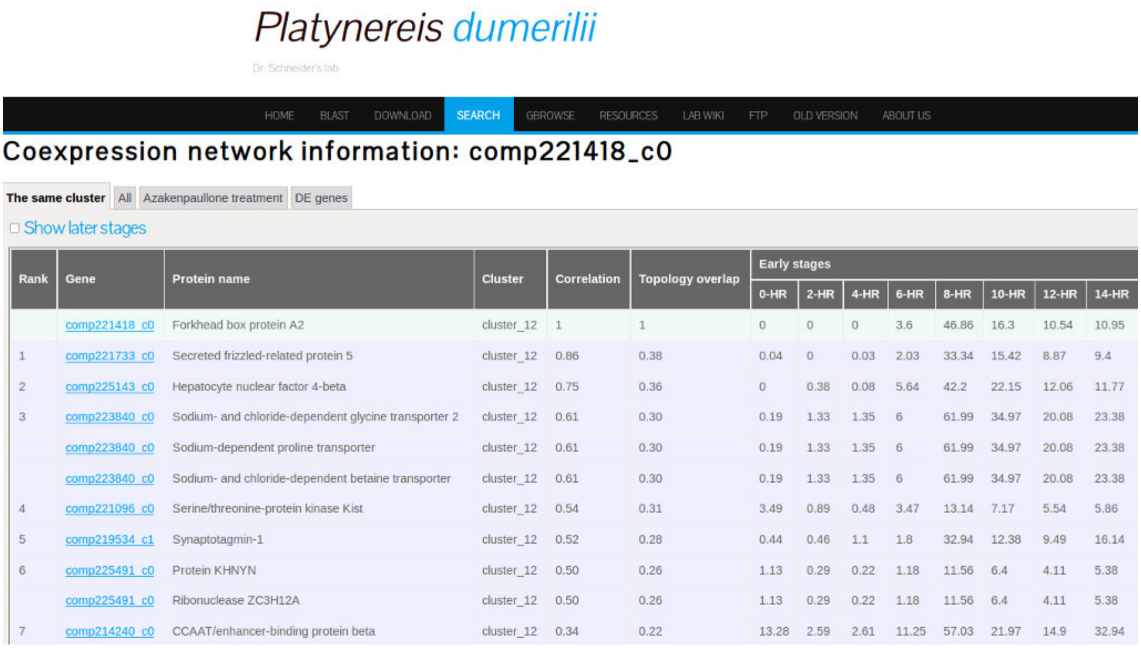

Rank	Gene	Protein name	Cluster	Correlation	Topology overlap	Early stages							
						0-HR	2-HR	4-HR	6-HR	8-HR	10-HR	12-HR	14-HR
	comp221418_c0	Forkhead box protein A2	cluster_12	1	1	0	0	0	3.6	46.86	16.3	10.54	10.95
1	comp221733_c0	Secreted frizzled-related protein 5	cluster_12	0.86	0.38	0.04	0	0.03	2.03	33.34	15.42	8.87	9.4
2	comp225143_c0	Hepatocyte nuclear factor 4-beta	cluster_12	0.75	0.36	0	0.38	0.08	5.64	42.2	22.15	12.06	11.77
3	comp223840_c0	Sodium- and chloride-dependent glycine transporter 2	cluster_12	0.61	0.30	0.19	1.33	1.35	6	61.99	34.97	20.08	23.38
	comp223840_c0	Sodium-dependent proline transporter	cluster_12	0.61	0.30	0.19	1.33	1.35	6	61.99	34.97	20.08	23.38
	comp223840_c0	Sodium- and chloride-dependent betaine transporter	cluster_12	0.61	0.30	0.19	1.33	1.35	6	61.99	34.97	20.08	23.38
4	comp221096_c0	Serine/threonine-protein kinase Kist	cluster_12	0.54	0.31	3.49	0.89	0.48	3.47	13.14	7.17	5.54	5.86
5	comp219534_c1	Synaptotagmin-1	cluster_12	0.52	0.28	0.44	0.46	1.1	1.8	32.94	12.38	9.49	16.14
6	comp225491_c0	Protein KHNYN	cluster_12	0.50	0.26	1.13	0.29	0.22	1.18	11.56	6.4	4.11	5.38
	comp225491_c0	Ribonuclease ZC3H12A	cluster_12	0.50	0.26	1.13	0.29	0.22	1.18	11.56	6.4	4.11	5.38
7	comp214240_c0	CCAAT/enhancer-binding protein beta	cluster_12	0.34	0.22	13.28	2.59	2.61	11.25	57.03	21.97	14.9	32.94

Figure 24: **PdumBase Search results interface** ”Show other info” option selected. The **Coexpression info** link displays the list of genes clustered with the gene under study.

Orthologs groups link

Clicking the Orthologs groups link gives access to an interface with tree tabs: List, Tree-ML and Tree-Parsimony. As mentioned in the section ”Comparative transcriptome data”, 18 species were selected to assess the ortholog groups. The first tab shows the list of species from which orthologs were found for the searched gene. This interface also allows to download the protein and cDNA sequences of the orthologs in Fasta format (see Figure 25).

The second and third tab under the Orthologs groups link will display phylogenetic trees based on ML and parsimony analysis respectively. Figure 26 shows the tree-ML for the FoxA2 ortholog genes.

Gene: comp221418_c0
Group: orth4921

List | Tree-ML | Tree-Parsimony

Species	Name	Class	Protein ID	Protein Sequence	cDNA Sequence
pdu	Platynereis dumerilii	Protostomia	comp221418_c0_seq9		
cte	Capitella teleta	Protostomia	estExt_Genewise1Plus.C_2250069		
hro	Helobdella robusta	Protostomia	147202		
lgi	Lottia gigantea	Protostomia	estExt_Genewise1.C_sca_70166		
cgi	Crassostrea gigas	Protostomia	EKC29500		
dpu	Daphnia pulex	Protostomia	gw1.69.46.1		
tca	Tribolium castaneum	Protostomia	TCOGS2:TC013245-PA		
dme	Drosophila melanogaster	Protostomia	FBpp0303365		
spu	Strongylocentrotus purpuratus	Deuterostomia	WHL22.439762.0		
sko	Saccoglossus kowalevskii	Deuterostomia	Sakowv30037646m		
bfo	Branchiostoma floridae	Deuterostomia	estExt_fgenesh2_pg.C_420051		
bfo	Branchiostoma floridae	Deuterostomia	fgenesh2_pm.scaffold_42000007		
dre	Danio rerio	Deuterostomia	ENSDARP00000002213		
dre	Danio rerio	Deuterostomia	ENSDARP00000007827		
xtr	Xenopus tropicalis	Deuterostomia	ENSXETP00000056642		
xtr	Xenopus tropicalis	Deuterostomia	ENSXETP00000026943		
hsa	Homo sapiens	Deuterostomia	ENSP00000250448		
hsa	Homo sapiens	Deuterostomia	ENSP00000400341		
nve	Nematostella vectensis	Nonbilateria	estExt_gwp.C_580130		
tad	Trichoplax adherens	Nonbilateria	TriadP27448		

Figure 25: **PdumBase Search results interface** ”Show other info” option selected. The **Ortholog groups** link displays the list species where orthologs were found. For FoxA2, orthologs were found in all of the 18 selected species.

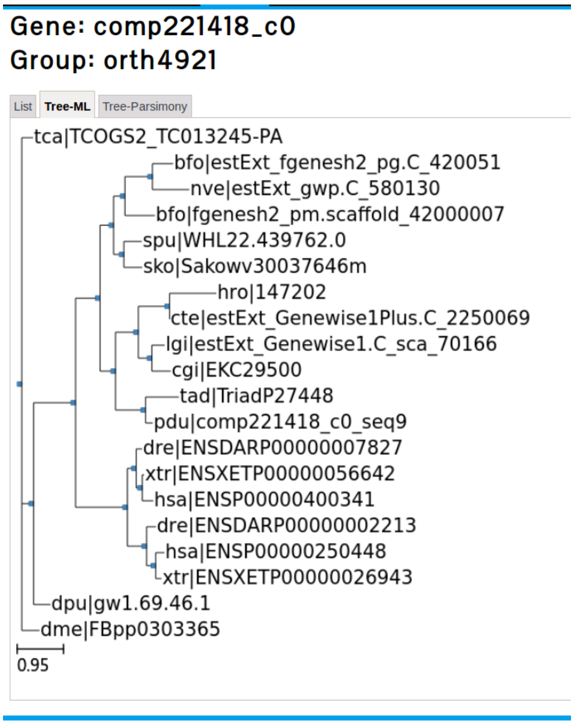

Figure 26: **PdumBase Search results interface** "Show other info" option selected. The **Ortholog groups** Tree-ML tab displays phylogenetic tree constructed with the ortholog protein sequences. Tree-ML for FoxA2 orthologs among the 18 species.

Example search for ”Homeobox genes”

This final example will show a sample search with multiple results, indicating the options that our web database offers to download the data in case further analysis is required.

Finding homeobox genes that are highest expressed at 8hpf

Searching for homeobox term in the blast field at the search interface will retrieve 114 hits. To find the highest expressed homeobox genes at 8 hpf, is is required to sort the hits by expression values at 8 hpf in descending order (See Figure 27).

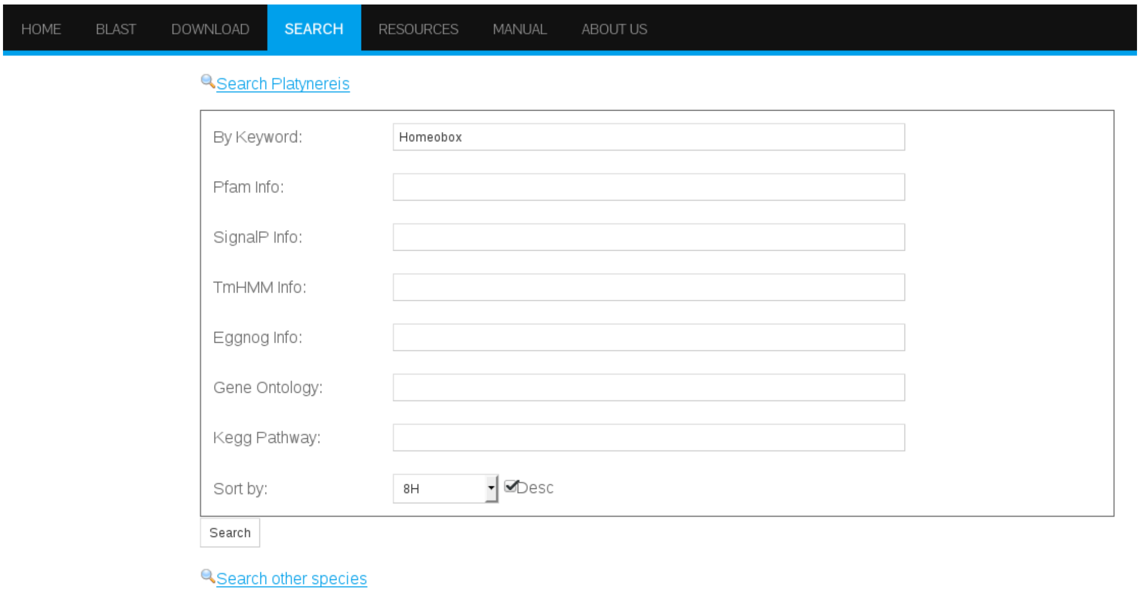

Figure 27: **PdumBase Search interface** search required to Find homeobox genes that are highest expressed at 8hpf.

Downloading results from *Platynereis dumerilii* web database

One important feature of our web database is that it allows to download the search results in different formats. The search results can be downloaded in both, comma separated value (CSV) format file and Excel file. Furthermore, the protein sequences from the genes displayed in the results can be downloaded in Fasta format. Links to download are found in the upper frame of the search result interface (see Figure 28).

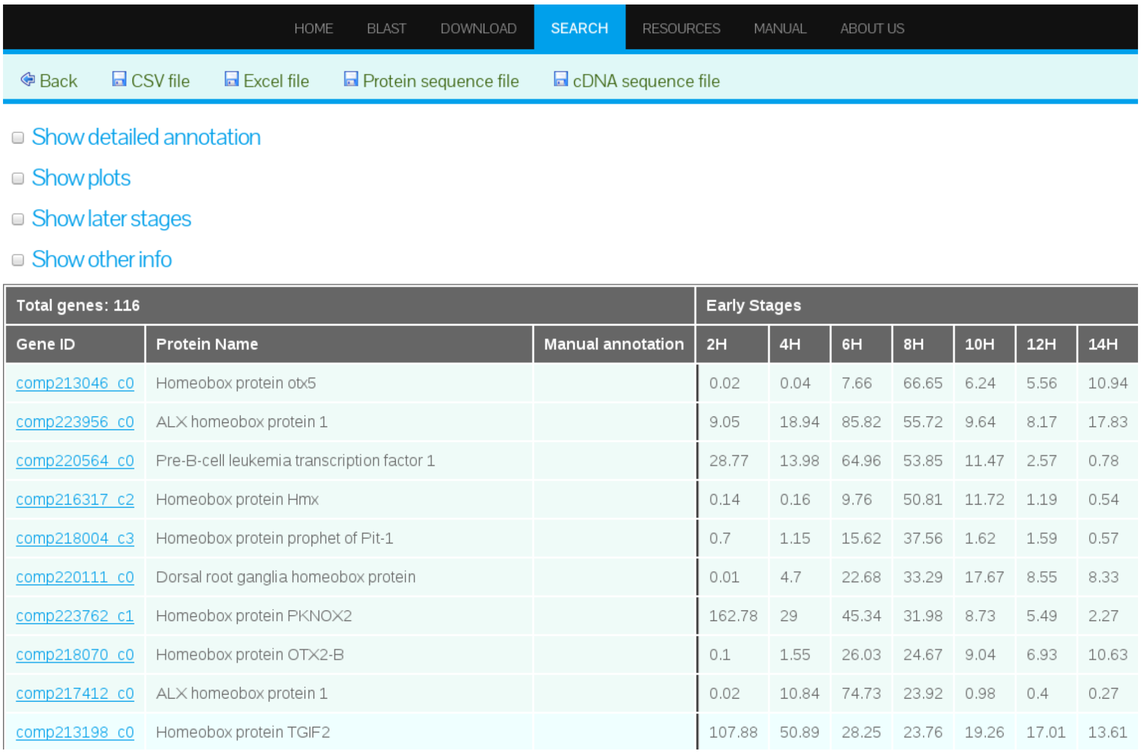

Total genes: 116			Early Stages						
Gene ID	Protein Name	Manual annotation	2H	4H	6H	8H	10H	12H	14H
comp213046_c0	Homeobox protein otx5		0.02	0.04	7.66	66.65	6.24	5.56	10.94
comp223956_c0	ALX homeobox protein 1		9.05	18.94	85.82	55.72	9.64	8.17	17.83
comp220564_c0	Pre-B-cell leukemia transcription factor 1		28.77	13.98	64.96	53.85	11.47	2.57	0.78
comp216317_c2	Homeobox protein Hmx		0.14	0.16	9.76	50.81	11.72	1.19	0.54
comp218004_c3	Homeobox protein prophet of Pit-1		0.7	1.15	15.62	37.56	1.62	1.59	0.57
comp220111_c0	Dorsal root ganglia homeobox protein		0.01	4.7	22.68	33.29	17.67	8.55	8.33
comp223762_c1	Homeobox protein PKNOX2		162.78	29	45.34	31.98	8.73	5.49	2.27
comp218070_c0	Homeobox protein OTX2-B		0.1	1.55	26.03	24.67	9.04	6.93	10.63
comp217412_c0	ALX homeobox protein 1		0.02	10.84	74.73	23.92	0.98	0.4	0.27
comp213198_c0	Homeobox protein TGIF2		107.88	50.89	28.25	23.76	19.26	17.01	13.61

Figure 28: **PdumBase Search results interface**. Here the result page is displaying the ten top hits, sorted by expression level at 8 hpf. Links to download data are shown with a floppy disk icon and are found in the upper frame.

Concluding Remarks

Given the here presented features and ease of use that our *Platynereis dumerilii* database offers, we are confident that this work will provide a reliable resource to the community for transcriptome studies due to its extensive content and user friendly design.

List of Figures

1 **PdumBase Search result interface** displays mean FPKM as measurement of absolute expression . . . 3

2 **PdumBase Expression data tab interface:** **(a)** The upper frame displays mean FPKM and raw counts data, from samples as a pool. **(b)** Lower frame displays expression data from individual replicas. . . . 4

3 **PdumBase Search results interface** displays Uniprot annotation data on the rightmost panel. Annotation data includes accession number, gene name, protein name, species and E-value. Clicking on the accession number will redirect to the UniProt page for that particular protein. . . . 4

4 **PdumBase Search results interface.** **(a)** The search result page allows to customize the information displayed by checking one or more options from the left top corner. **(b)** Selecting the option: **"Show detailed annotation"** will show detailed gene ontology, KEGG Pathways and protein domain annotation. . . . 5

5 **PdumBase Annotation tab interface.** This tab is available once an entry from the result page has been selected. It is accessible via result page → clicking on a gene or transcript of interest → clicking on the Annotation tab . . . 7

6 **PdumBase Search result interface.** Expression profile plots are displayed when the option **"Show plots"** is selected. . . . 8

7 **PdumBase Plot tab interface**. Shows the expression profile plot for a given transcript. . . . 9

8 Heat map of 13,160 expressed genes clustered into 15 groups according to the time series patterns. . . . 9

9 **PdumBase Coexpression information interface**. Displays all the transcripts/genes in the same cluster of a given component, shows protein name, correlation and topology overlap. . . . 10

10 **PdumBase Ortholog expression profile interface**. Displays the expression data from the selected *Platynereis dumerilii* gene and the orthologs genes found in the other species along with their expression and annotation data (when available). . 11

11 **PdumBase List tab interface** under Ortholog groups. Shows the species list, code, name, ortholog protein ID and contains links to access/download the protein and cDNA sequences in Fasta format. . . . 13

12 **PdumBase Ortholog groups interface:** **(a)** Phylogenetic tree among ortholog genes displayed under **Tree-ML tab** **(b)** Phylogenetic tree displayed under **Tree-Parsimony tab**. Both trees show the species code and the transcript/gene ID. . . 14

13 **PdumBase Alignment tab interface** under Ortholog groups. Displays CLUSTAL 2.1 multiple sequence alignment. . . . 14

14 **PdumBase Search interface**. . . . 15

15 **PdumBase Search interface**. Searching for FoxA2 . . . 16

16 **PdumBase Search result interface**. shows Gene ID, expression data from early stages and from inhibitor experiment, and annotation information. The data retrieve options are found on the left upper corner. . . . 16

17 **PdumBase Plot tab** from Detailed data results interface. Displaying expression profile plots for FoxA2. . . . 17

18 **PdumBase Expression data tab** from Detailed data results interface. **(a)** Displays expression data (mean FPKM and raw counts) from pooled samples from early stages of development. **(d)** show individual replicates expression data for early stages 18

19 **PdumBase Annotation tab** from Detailed data results interface. Displaying detailed annotation information for FoxA2. . . . 19

20 **PdumBase Search results interface** checking the boxes from the search result options on the left will expand the results displayed. 20
21 **PdumBase Search results interface** with the option "Show Plots" selected. Expression plots for both, early and late stages are shown for the gene under search: FoxA2. 20
22 **PdumBase Search results interface** with the option "Show later stages" selected. Here the later stages expression data from FOXA2 is displayed. 21
23 **PdumBase Search results interface** "Show other info" option selected. Additional information links are displayed. 21
24 **PdumBase Search results interface** "Show other info" option selected. The **Coexpression info** link displays the list of genes clustered with the gene under study. 22
25 **PdumBase Search results interface** "Show other info" option selected. The **Ortholog groups** link displays the list species where orthologs were found. For FoxA2, orthologs were found in all of the 18 selected species. 23
26 **PdumBase Search results interface** "Show other info" option selected. The **Ortholog groups** Tree-ML tab displays phylogenetic tree constructed with the ortholog protein sequences. Tree-ML for FoxA2 orthologs among the 18 species. . . 24
27 **PdumBase Search interface** search required to Find homeobox genes that are highest expressed at 8hpf. 25
28 **PdumBase Search results interface**. Here the result page is displaying the ten top hits, sorted by expression level at 8 hpf. Links to download data are shown with a floppy disk icon and are found in the upper frame. 26

List of Tables

1 Time points from Early Stages data set 2
2 Late Stages included in data set. Time points are shown in hours post fertilization (hpf), days (d) and months (M). 3
3 Species and number of protein sequences for comparative analysis 11
4 Number of orthologs genes between the 6 species 12
5 Species and number of genes used to find orthologs groups 12
